# Male Genitalia of Neotropical Charaxinae: A Comparative Analysis of Character Variation

**DOI:** 10.1673/031.013.3501

**Published:** 2013-04-18

**Authors:** Dayana Bonfantti, Mirna Martins Casagrande, Olaf Hermann Hendrik Mielke

**Affiliations:** 1 Departamento de Zoologia; Setor de Ciências Biológicas; Universidade Federal do Paraná; C. P.: 19020; 81531980, Curitiba, Paraná, Brazil

**Keywords:** Anaeini, Anaeomorphini, butterflies, morphology, Preponini

## Abstract

Charaxinae (Lepidoptera: Nymphalidae) has a worldwide distribution, although it occurs mainly between the tropics. Most species occur in the Neotropics, where three tribes, Preponini, Anaeomorphini, and Anaenini, can be found. Collectively, these three tribes encompass 109 species. Because of its relevance to systematics and taxonomy, the male genitalia of Lepidoptera have been extensively studied. The male genitalia are composed of the last two abdominal segments and their modifications for mating, known as claspers of the bodies. In order to improve upon the systematic classification of the subfamily, 31 species of 13 genera of Neotropical Charaxinae were analyzed. All characters relevant to species and generic taxonomy were analyzed. Most structures showed morphological variations among tribes, genera, and species. These variations demonstrated to be important to Preponini, because the structural patterns of the genitalia allow the separation in two groups, *Prepona* Boisduval and *Archaeoprepona* Fruhstorfer, and are in accord with the recent taxonomic classification proposed by Ortiz-Acevado and Willmott (2013), wherein *Agrias* Doubleday is synonymized in *Prepona* and *Noreppa* Rydon within *Archaeoprepona.* In the same way, *Anaeomorpha splendida* Rothschild showed considerable differences from Preponini's genera, the tribe in which it was included, confirming the revalidation of the tribe Anaeomorphini (Ortiz-Acevado and Willmott 2013). Substantial variation was found in the genital structures of Anaeini, making it difficult to establish structural patterns for this group. Such structural variation, however, may be very efficient to diagnose species, such as some species of *Memphis* Hübner and *Fountainea* Rydon, which can be easily identified through the presence and location of spines on the valva.

## Resumo

Charaxinae (Lepidoptera: Nymphalidae) tem distribuição mundial, embora ocorra principalmente entre trópicos. A maioria das espécies distribui-se na região Neotropical, onde três tribos podem ser encontradas, Preponini, Anaeomorphini, e Anaeini. Coletivamente, estas tribos abrangem 109 espécies. Devido à sua relevância para a sistemática e taxonomia, a genitália masculina de Lepidoptera, formada pelos dois últimos segmentos abdominais, tem sido extensivamente estudada. Com a finalidade de aperfeiçoar a classificação sistemática da subfamília, foram analisadas 31 espécies de 13 gêneros de Charaxinae neotropicais. A maioria das estruturas apresentou variações morfológicas entre as tribos, gêneros e espécies. Estas variações demostraram ser importantes para Preponini, porque os padrões estruturais das genitálias permitiram que fossem separados em dois grupos, o primeiro *Prepona* Boisduval e o segundo *Archaeoprepona* Fruhstorfer além de estar de acordo com a recente classificação taxonômica proposta por Ortiz-Acevado and Willmott (2013), em que *Agrias* Doubleday esta sinonimizada em *Prepona* e *Noreppa* Rydon em *Archaeoprepona.* Da mesma maneira, *Anaeomorpha splendida* Rothschild apresentou diferenças consideráveis em relação aos gêneros de Preponini, tribo na qual estava incluída, confirmando a revalidação da tribo Aneomorphini (Ortiz-Acevado and Willmott 2013). Variações substanciais nas estruturas genitais de Anaeini foram encontradas, tornando-se dificil estabelecer padrões para esta tribo, embora tais variações estruturais possam ser muito eficientes para diagnosticar espécies, tais como algumas espécies de *Memphis* Hübner e *Fountainea* Rydon, facilmente identificadas através da presença e localização de espinhos na valva.

## Introduction

The male genitalia of butterflies has been the subject of several very detailed studies, as its morphology can provide the basis for work of taxonomy, systematic analysis, and phylogenetic analysis (Simonsen 2006a). Some recent work on the comparative morphology of male genitalia has examined genital muscles (Simonsen 2006a, b) and intraspecific structural variations (Caldas 1997; Tóth 2011).

Charaxinae (Lepidoptera: Nymphalidae) are a group of medium to large, robust butterflies. They fly fast and circulate around tree canopies, behaviors that make them difficult to observe. Males and females are easily attracted by bait containing fruit, decomposing animals, feces, urine, and/or sweat (Rydon 1971). Charaxinae butterflies occur mainly between the tropics, primarily inhabiting the Neotropical region (Mielke et al. 2004a).

Recently, this subfamily has undergone drastic changes in its taxonomic classification. Through phylogenetic study of the tribe Preponini, using data from DNA sequences, Ortiz-Acevado and Willmott (2013) classified Preponini as follows: two genera were synonymized, *Noreppa* to *Archaeoprepona* and *Agrias* to *Prepona*; the fifth genus (monotypic), *Anaeomorpha splendida,* had its tribe status revalidated to Anaeomorphini. So, the neotropical Charaxinae contained two tribes, 17 genera, and 109 species (Salazar and Constantine, 2001; Lamas, 2004; Salazar, 2008), and today is made up of three tribes, 15 genera, and 109 species (Ortiz-Acevado and Willmott 2013).

The Charaxinae are characterized by the following synapomorphies: parapatagium sclerotized (Ehrlich 1958); forewings with R_4_ and R_5_ longer than their common branch; R_4_ curved downward near its end, which may be at the costal margin, the apex, or the outer margin of the wing; posterior discal cell closed; anepisternum II present as a distinct sclerite; and mesothoracic pre-episternum well-developed (Ackery 1984). These synapomorphies were corroborated in a series of contributions by Mielke et al. (2004a, b, c), who also found some variation in the way vein R_4_ ends in *Zaretis itys itylus* (Westwood) and *Prepona claudina annetta* (Gray).

With the goal to clarify the classification and systematics of the Neotropical Charaxinae, we have illustrated, compared, and ascertained variation in the male genitalia of 31 species of 13 genera. Some of the characters mentioned have been previously mentioned in other literature, whereas others are new.

## Materials and Methods

The species studied and material studied are listed in Table 1. The specimens analyzed are retained at Coleção Entomológica Pe. Jesus Santiago Moure, Departamento de Zoologia, Universidade Federal do Paraná, and Coleção Prof. Dr. Olaf Mielke.

The abdomen of each exemplar was removed and immersed in potassium hydroxide 10% for at least 24 hours (depending on the size of the abdomen) to soften the tissues. After that, the abdomen was washed in water.

The genitalia were removed through a slit on the pleura of the 7^th^ abdominal segment. After study, the genital parts were stored in a microvial containing glycerin, which was attached to the same pin as the exemplar.

Drawings were made using a camera lucida attached to a dissecting microscope. The main lines were inked and watercolor was used to mark each sclerite with a different color.

Illustrations were coded as follows: full lines represent the boundaries of morphological structures on the first focal plane; dashed lines represent the boundaries of structures that lay underneath the main structure under study. As with structures represented by dotted lines, structures represented by dashed lines were only shown when they were important for the interpretation of the genital anatomy. Sclerites lacking a well-defined boundary were represented by intermediate colors. Sclerites, and their respective color codes, are as follows: tegumen - yellow; saccus - cadmium red; uncus - navy blue; gnathos - alizarin red; fultura inferior - turquoise; valvae - lemon yellow; penis - green.

In some illustrations, the sclerites were omitted in order to facilitate interpretation. For instance, the fultura inferior was omitted from illustrations showing the lateral aspect of the genitalia. A minimum of nine drawings were produced for each species as follows: lateral, dorsal, and posterior aspects of the genitalia; inner and outer aspects of the valva; and lateral (left and right), dorsal, and ventral aspects of the penis. In the descriptions and comparisons, only the greatest values are given for width and length.

SEM photographs were taken following standard procedures. First, the parts were dehydrated by immersion in decreasing ethanol dilutions (70%, 80%, 90%, and 100%) for 10 minutes, and then immersed for an additional 10 minutes in ethanol absolute. Second, they were subjected to critical point drying, glued to a stub, and coated.

**Figure 1.**
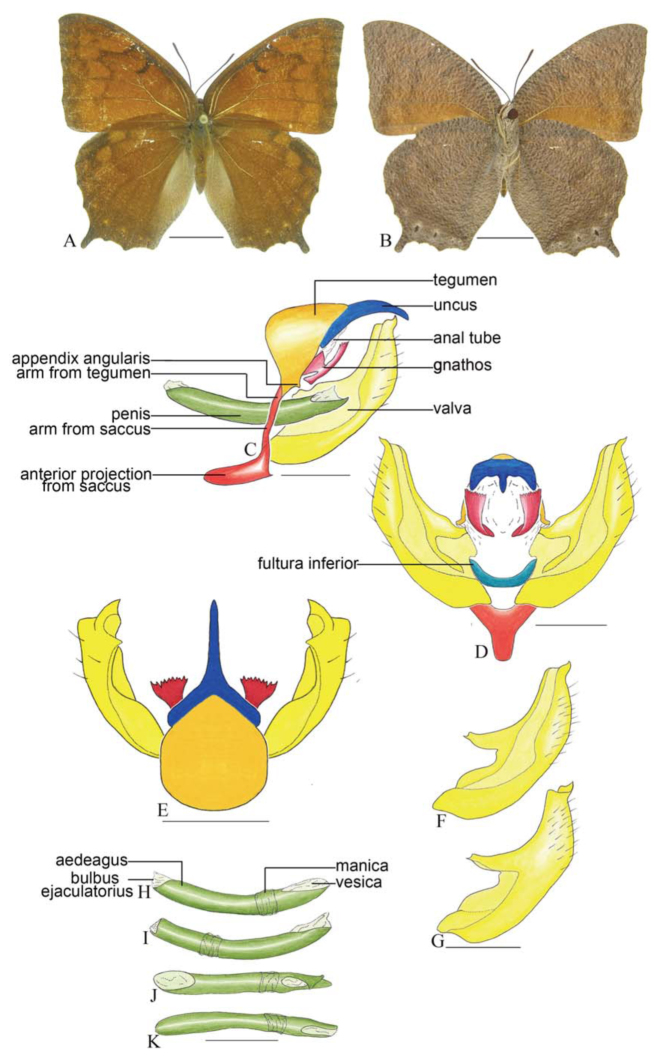
*Anaea troglodyta* (Fabricius). A. Dorsal. B. Ventral. C–K Genitalia: C. lateral view. D. Posterior view. E. Dorsal view. F and G Valva: F. Internal. G. External. H–K: Penis: H. Right lateral view. I. Left lateral view. J. Dorsal view. K. Ventral view. Scale bar: A and B = 1 cm. C–K = 1 mm. High quality figures are available online.

The main terminology used was adopted from Niculescu (1978) (Figure 1), with adaptations from Petersen (1904), Pierce (1909; 1914), Kuznetsov (1915), Snodgrass (1935), Oiticica (1946), Okagaki et al. (1954), Sibatani et al. (1954), Klots (1956), Ogata et al. (1957), Snodgrass (1957), Srivastava (1965), Casagrande (1979), and Bilotta (1994).

## Results

### Anaeini

***Anaea troglodyta* (Fabricius) (Figure 1 A– K).** Tegumen triangular in lateral view, anterior projection and appendices angulares wider than long. Arms from tegumen and saccus distinct. Anterior projection from saccus elongated in lateral view; posterior projection shorter than anterior.

Uncus slender, with distal portion descending, in dorsal view fused with tegumen, uncus differing from tegumen by a light sclerotization. Anal tube reduced and entirely membranous. Gnathos open, in lateral view forming two distinct plates, with distal portion flattened on anterior ventral portion, gnathos extended dorsoventrally toward the proximal portion, with spines of various sizes that give the dorsal distal section a serrated aspect.

Valva subtriangular, with inner ends expanded. Fultura inferior forming a semicircle.

Penis: aedeagus sclerotized, with bulbus ejaculatorius projecting from antero-dorsal opening

**Figure 2.**
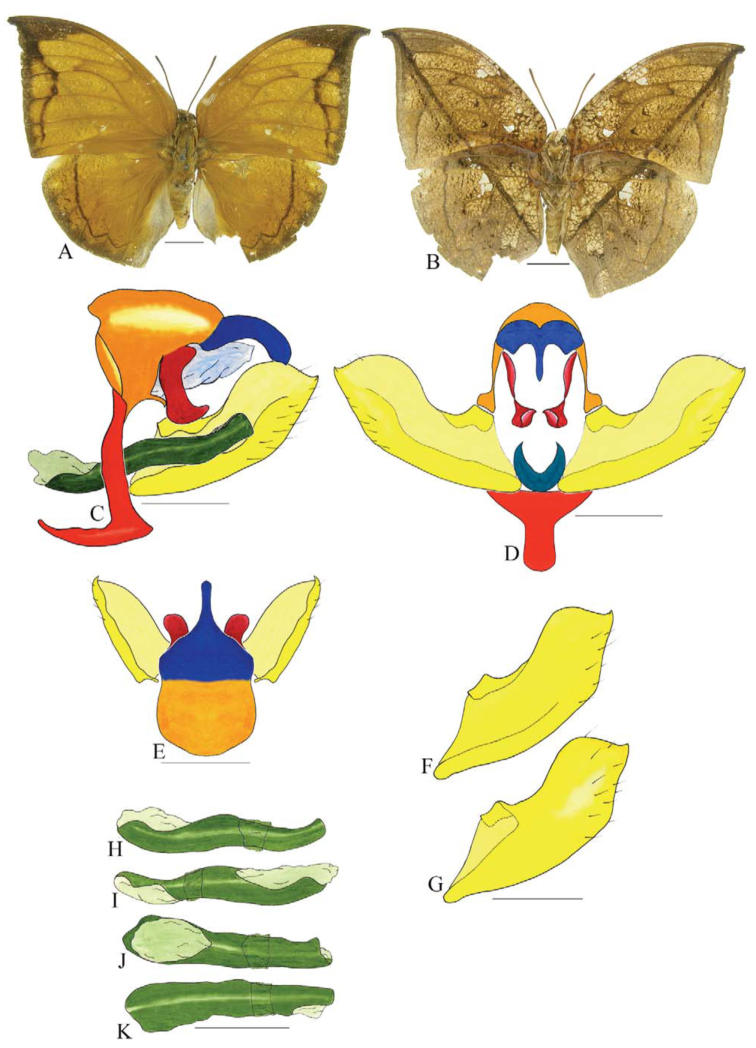
*Coenophlebia archidona* (Hewitson). A. Dorsal. B. Ventral. C–K Genitalia: C. Lateral view. D. Posterior view. E. Dorsal view. F and G Valva: F. Internal. G. External. H–K: Penis: H. Right lateral view. I. Left lateral view. J. Dorsal view. K. Ventral view. Scale bar: A and B = 1 cm. C–K = 1 mm. High quality figures are available online.

***Coenophlebta archidona* (Hewitson) (Figure 2 A–K).** Tegumen subtriangular in lateral view, anterior projection wider than long, tegument fold projecting outward from median to ventral region, appendices angulares longer than wide. Arms from tegumen and saccus distinct. Anterior projection from saccus elongate in lateral view; posterior projection absent.

Uncus thick, with distal portion descending, in dorsal view fused with tegumen, uncus differing from tegumen by a light sclerotization. Anal tube developed, with ventral portion sclerotized (subscaphium). Gnathos open, in lateral view with proximal portion entirely fused with tegumen, C-shaped, in posterior view curved with distal portion descending.

Valva subrectangular, with a dorso-proximal notch and a small spine on distal portion. Fultura inferior bilobed, V-shaped, with a deep dorsal notch.

Penis: aedeagus sinuous and completely sclerotized, with bulbus ejaculatorius projecting from antero-dorsal opening.

**Figure 3.**
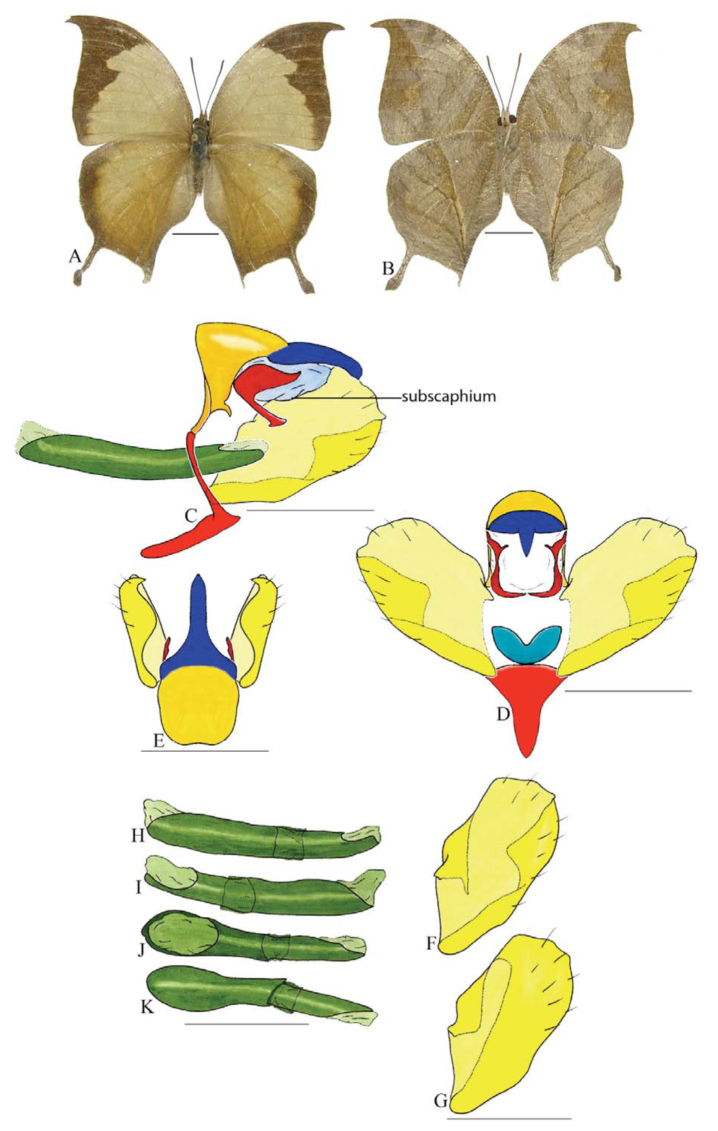
*Consul electro* (Westwood). A. Dorsal. B. Ventral. C–K Genitalia: C. Lateral view. D. Posterior view. E. Dorsal view. F and G Valva: F. Internal. G. External. H–K: Penis: H. Right lateral view. I. Left lateral view. J. Dorsal view. K. Ventral view. Scale bar: A and B = 1 cm. C–K = 1 mm. High quality figures are available online.

***Consul electra* (Westwood) (Figure 3 A–K).** Tegumen subtriangular in lateral view, anterior projection wider than long, tegument fold projecting outward from anteromedian portion of arms from tegument to their apex, appendices angulares longer than wide. Arms from tegumen and saccus distinct. Anterior projection from saccus elongate in lateral view, about twice as long as wide; posterior projection of saccus present, shorter than anterior projection.

Uncus slender, with distal portion descending, in dorsal view fused with tegumen, uncus differing from tegumen by a light sclerotization. Anal tube developed, with ventral portion sclerotized (subscaphium). Gnathos open, in lateral view with the distal portion of each gnathos close to each other, with two parts, one dorsal and one ventral distal, ventral part more developed and subrectangular; ventral distal part elongate and with a small descending apical club; in posterior view L-shaped aspect, expanded apically.

Valva rounded, inner tegument expanded over outer tegument on ventral distal portion. Fultura inferior bilobed and linked ventrally with the diaphragma rather than with the valva.

Penis: aedeagus sclerotized, expanded ventrally up to insertion of manica, and bulbus ejaculatorius projecting from antero-dorsal opening.

**Figure 4.**
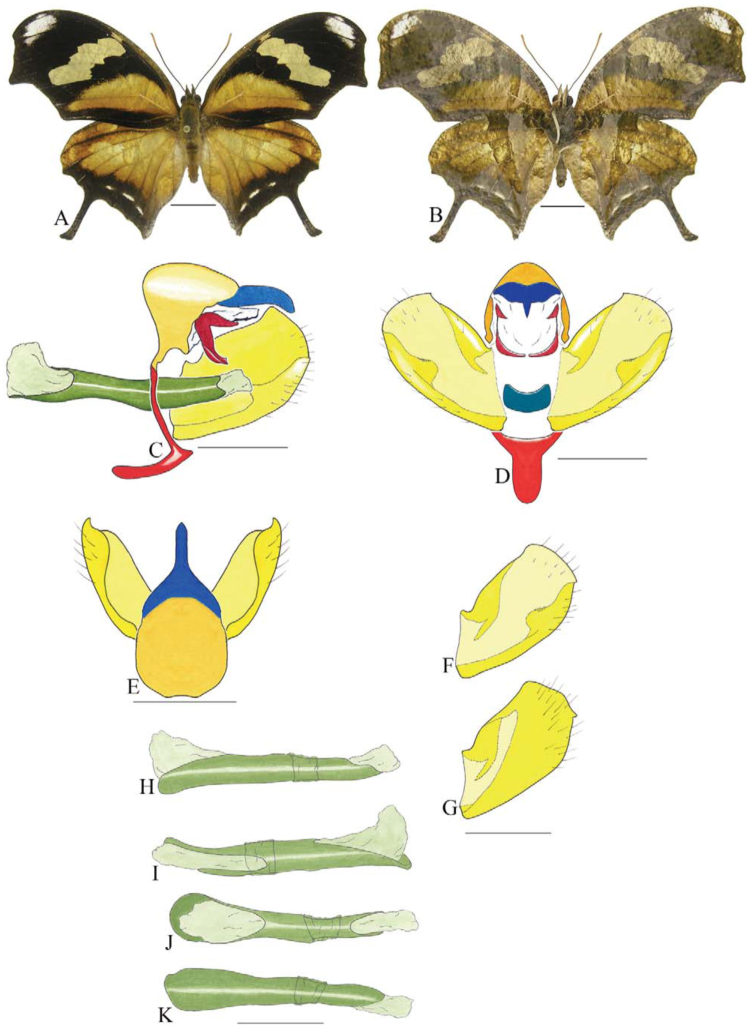
*Consul fabius drurii* (Butler). A. Dorsal. B. Ventral. C–K Genitalia: C. Lateral view. D. Posterior view. E. Dorsal view. F and G Valva: F. Internal. G. External. H–K: Penis: H. Right lateral view. I. Left lateral view. J. Dorsal view. K. Ventral view. Scale bar: A and B = 1 cm. C–K = 1 mm. High quality figures are available online.

***Consul fabius drurii* (Butler) (Figure 4 A–K, 33 C, D).** Tegumen subtriangular in lateral view; anterior projection and appendices angulares wider than long. Arms from tegumen and saccus distinct. Anterior projection from saccus elongated in lateral view, at least three times longer than wide; posterior projection present, shorter than anterior projection.

Uncus slender, with distal portion descending, in dorsal view fused with tegumen, uncus differing from tegumen by a light sclerotization. Anal tube developed, with ventral portion sclerotized (subscaphium), enveloped by the gnathos ventrally. Gnathos open, in lateral view with two parts: dorsal and ventral-distal, both elongated, ventral-distal part descending, with a slight fold in the tegument.

Valva approximately rounded; inner tegument expanded over outer tegument on ventral distal portion and bearing small spines on the harpe. Fultura inferior bilobed.

Penis: aedeagus completely sclerotized, with bulbus ejaculatorius projecting from anterodorsal opening, posterior opening large, originating underneath the manica.

**Figure 5.**
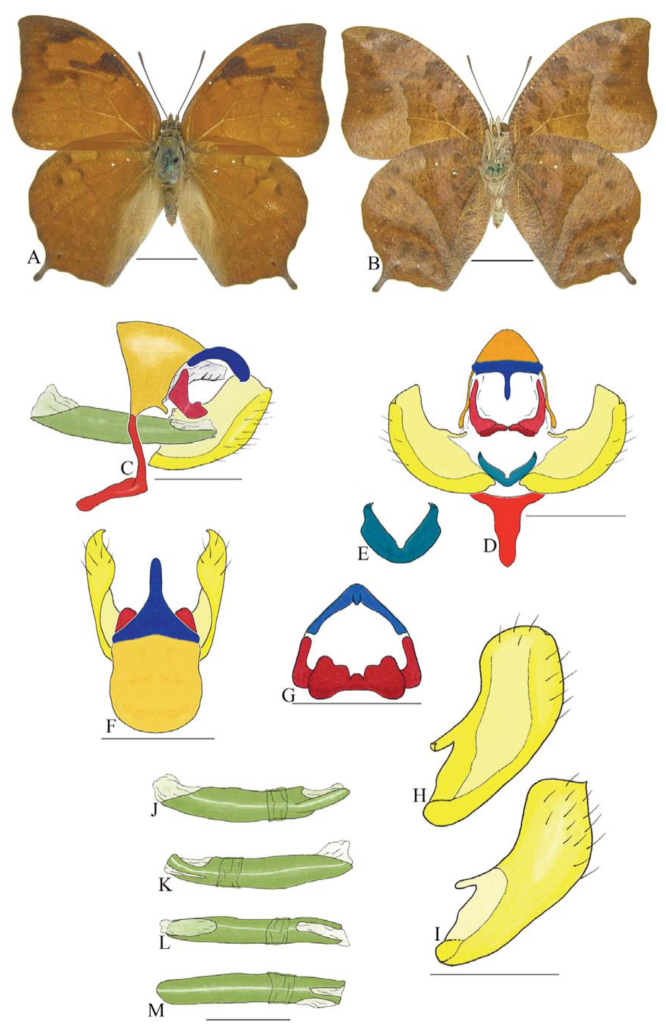
*Fountainea glycerium cratais* (Hewitson). A. Dorsal. B. Ventral. C–M Genitalia: C. Lateral view. D. Posterior view. E. Fultura inferior. F. Dorsal view. G. Gnathos. H and I Valva: H. Internal. I. External. J–M: Penis: J. Right lateral view. K. Left lateral view. L. Dorsal view. M. Ventral view. Scale bar: A and B = 1 cm. C–M = 1 mm. High quality figures are available online.

***Fountainea glycerium cratais* (Hewitson) (Figure 5 A–M).** Tegumen subtriangular in lateral view; without anterior projection, appendices angulares longer than wide. Arms from tegumen and saccus distinct. Anterior projection from saccus elongated in lateral view, at least three times longer than wide; posterior projection absent.

Uncus slender, apex slender and descending, in dorsal view fused with tegumen, uncus differing from tegumen by a light sclerotization. Anal tube developed, with ventral portion sclerotized (subscaphium) and enveloped by the gnathos ventrally. Gnathos closed and distally forming a round plate, projecting posteriorly.

Valva subrectangular and outer tegument forming a distal spine. Fultura inferior bilobed, with medium ventral notch, and small dorsal spines.

Penis: aedeagus sclerotized, bulbus ejaculatorius projecting from antero-dorsal opening; posterior opening irregular, with deep notches.

**Figure 6.**
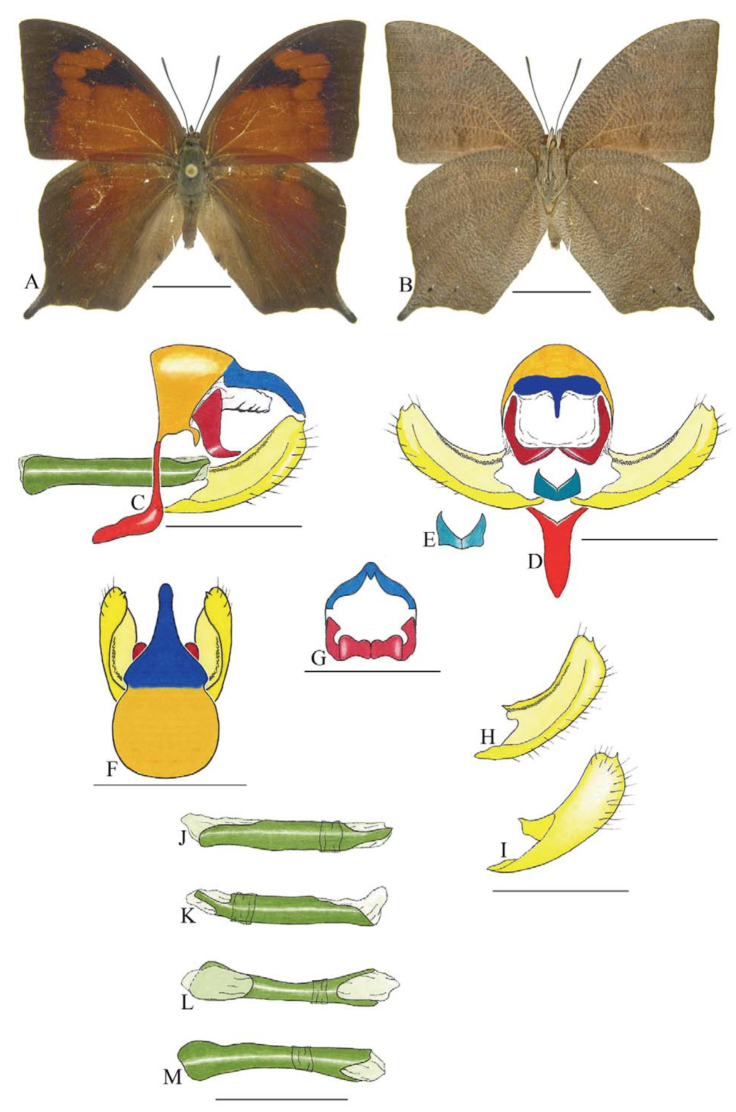
*Fountainea halice halice* (Godart). A. Dorsal. B. Ventral. C–M Genitalia: C. Lateral view. D. Posterior view. E. Fultura inferior. F. Dorsal view. G. Gnathos. H and I Valva: H. Internal. I. External. J–M: Penis: J. Right lateral view. K. Left lateral view. L. Dorsal view. M. Ventral view. Scale bar: A and B = 1 cm. C–M = 1 mm. High quality figures are available online.

***Fountainea halice halice* (Godart) (Figure 6 A–M).** Tegumen subtriangular in lateral view, without anterior projection, appendices angulares longer than wide. Arms from tegumen and saccus distinct. Anterior projection from saccus elongated in lateral view, at least twice longer than wide; posterior projection absent.

Uncus slender, with proximal ventral expansion and distal portion descending, in dorsal view fused with tegumen, uncus differing from tegumen by a light sclerotization. Anal tube developed, with ventral portion sclerotized (subscaphium) and enveloped by the gnathos ventrally. Gnathos closed, in lateral view sub rectangular, with anterior and posterior margins sinuous and fused medially

Valva subrectangular, with small spines near dorsal region and a distal spine. Fultura inferior bilobed, formed by two plates joined medially.

Penis: aedeagus sclerotized, with bulbus ejaculatorius projecting from antero-dorsal opening.

**Figure 7.**
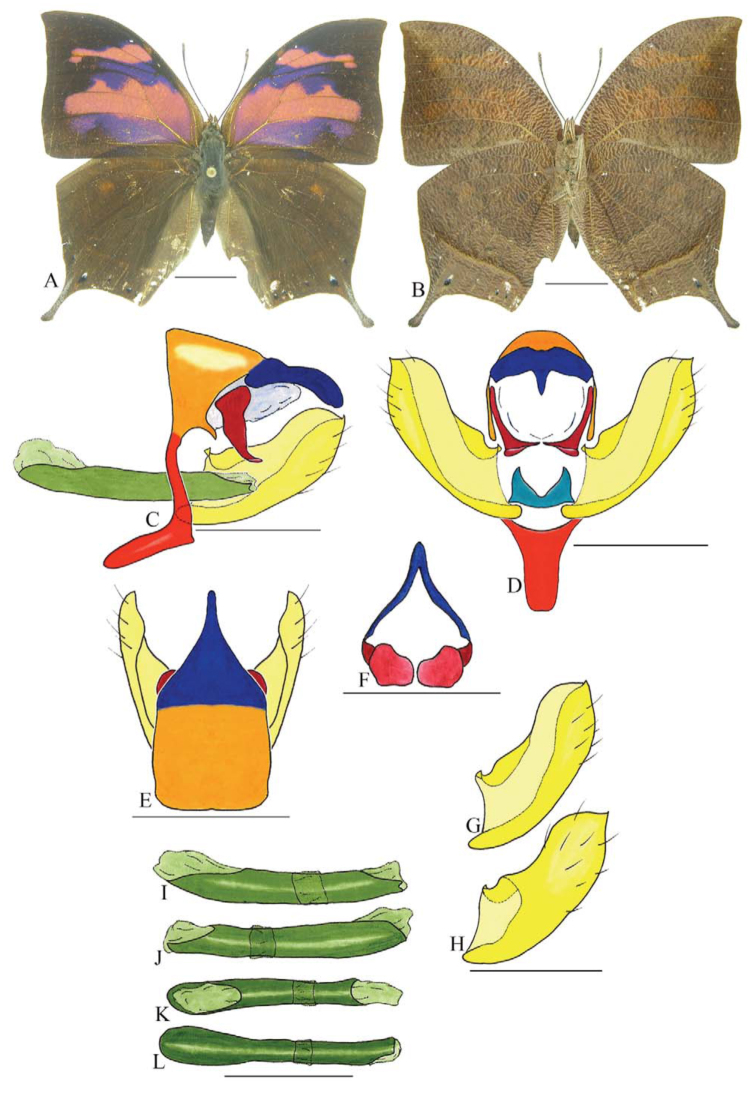
*Fountainea nessus* (Latreille). A. Dorsal. B. Ventral. C–L Genitalia: C. Lateral view. D. Posterior view. E. Dorsal view. F. Fultura inferior. G and H Valva: G. Internal. H. External. I–L Penis: I. Right lateral view. J. Left lateral view. K. Dorsal view. L. Ventral view. Scale bar: A and B = 1 cm. C–L = 1 mm. High quality figures are available online.

***Fountainea nessus* (Latreille) (Figure 7 A– L).** Tegumen subtriangular in lateral view, without anterior projection, appendices angulares longer than wide. Arms from tegumen and saccus fused. Anterior projection from saccus elongated in lateral view, at least three times longer than wide; posterior projection absent.

Uncus slender, basal portion enlarged, with distal portion descending, in dorsal view fused with tegumen, uncus differing from tegumen by a light sclerotization. Anal tube developed and entirely membranous. Gnathos open, in lateral view rounded and with distal portion of both gnathos close to each other, differing from the remaining species of *Fountainea.*

Valva subrectangular, bearing distal spine. Fultura inferior large and bilobed.

Penis: aedeagus sclerotized, with bulbus ejaculatorius projecting from antero-dorsal opening, posterior opening sinuous.

**Figure 8.**
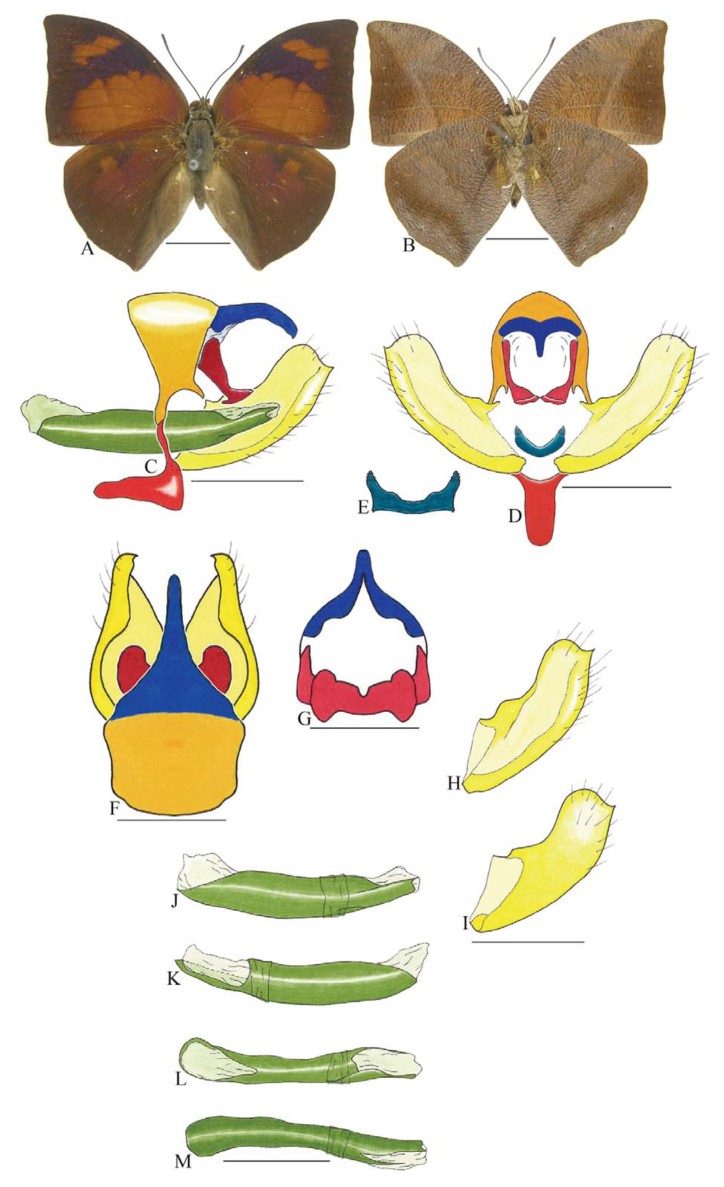
*Fountainea ryphea phidile* (Geyer). A. Dorsal. B. Ventral. C–M Genitalia: C. Lateral view. D. Posterior view. E. Fultura inferior. F. Dorsal view. G. Gnathos. H and I Valva: H. Internal. I. External. J–M: Penis: J. Right lateral view. K. Left lateral view. L. Dorsal view. M. Ventral view. Scale bar: A and B = 1 cm. C–M = 1 mm. High quality figures are available online.

***Fountainea ryphea phidile* (Geyer) (Figure 8 A–M, 33 A, B).** Tegumen subtriangular in lateral view, anterior projection wider than long, appendices angulares longer than wide. Arms from tegumen and saccus distinct. Anterior projection from saccus elongated in lateral view, at least twice as long as wide; posterior projection absent.

Uncus slender, with distal portion descending, in dorsal view fused with tegumen, uncus differing from tegumen by a light sclerotization. Anal tube reduced and entirely membranous. Gnathos closed, in lateral view forming a single round plate, similar to the gnathos of *F. glycerium cratais.*

Valva subrectangular, bearing distal spine. Fultura inferior wide and semi-circular, with reduced spines on apical dorsal tips.

Penis: aedeagus sclerotized, with bulbus ejaculatorius projecting from antero-dorsal opening.

**Figure 9.**
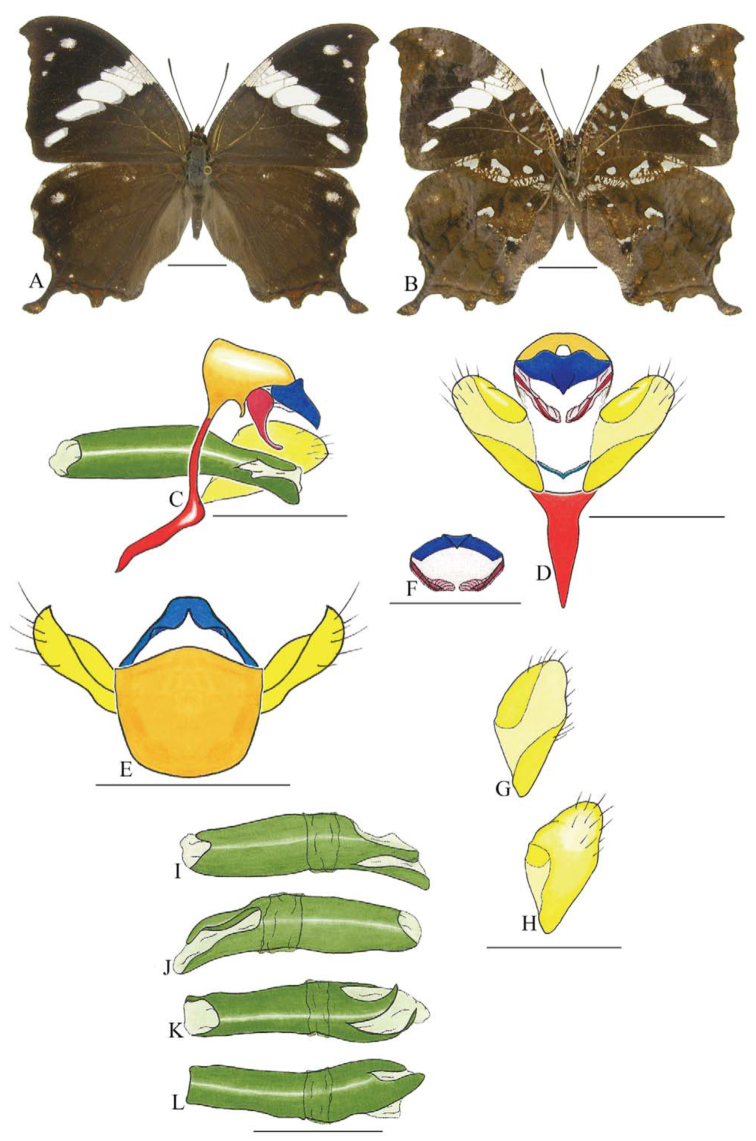
*Hypna clytemnestra forbesi* Godman and Salvin. A. Dorsal. B. Ventral. C–L Genitalia: C. Lateral view. D. Posterior view. E. Dorsal view. F. Fultura inferior. G and H Valva: G. Internal. H. External. I–L Penis: I. Right lateral view. J. Left lateral view. K. Dorsal view. L. Ventral view. Scale bar: A and B = 1 cm. C–L = 1 mm. High quality figures are available online.

***Hypna clytemnestra forbesi* Godman and Salvin (Figure 9 A–L).** Tegumen subtraprezoidal in lateral view, anterior projection wider than long, appendices angulares longer than wide. Arms from tegumen and saccus fused. Anterior projection from saccus elongated in lateral view, at least four times longer than wide, apex slender and curved; posterior projection absent.

Uncus thick, dorsal proximal portion crest-like, apex slender and descending, in dorsal view not completely fused with tegumen, uncus with a basal fenestra, with two dorsal distal lobes. Anal tube reduced and entirely membranous. Gnathos open, in lateral view apostrophe-like, proximal portion underneath tegumen, outer distal portion descending and curved, ventral portion elongated, descending, with distal portions of both gnathos close to each other and enveloped in membranes.

Valva approximately semi-circular. Fultura inferior reduced, V-shaped.

Penis: aedeagus sclerotized and sinuous, with bulbus ejaculatorius projecting from anterodorsal opening.

**Figure 10.**
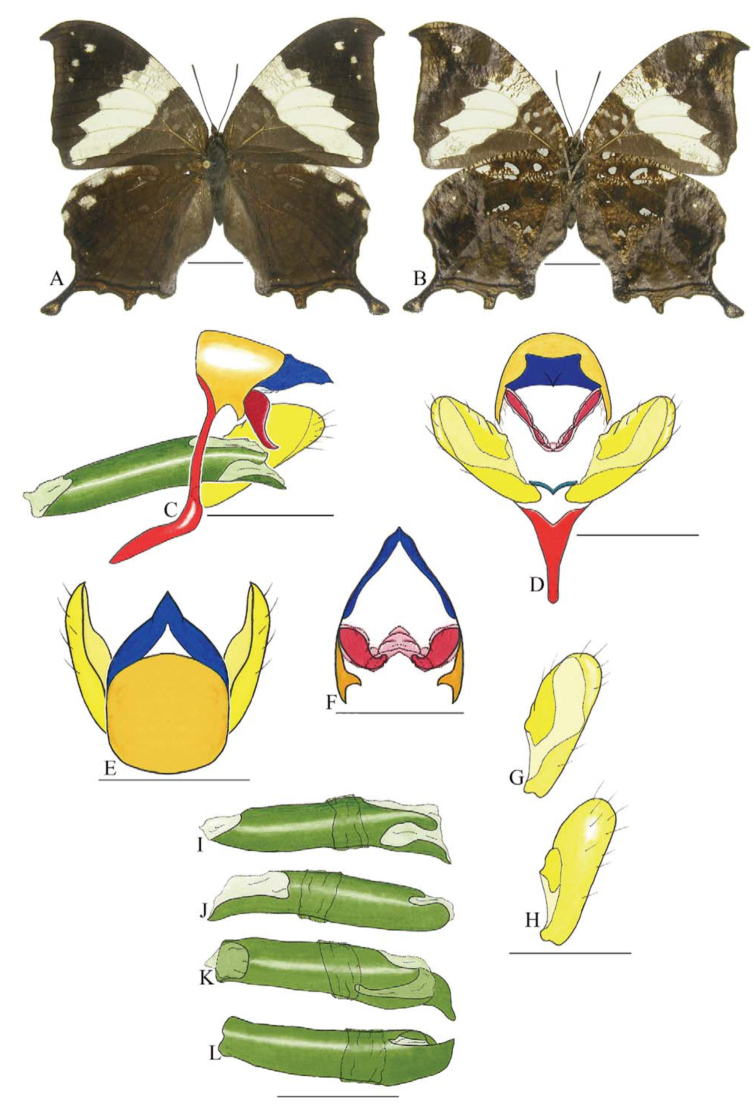
*Hypna clytemnestra huebneri* Butler. A. Dorsal. B. Ventral. C–L Genitalia: C. Lateral view. D. Posterior view. E. Dorsal view. F. Fultura inferior. G and H Valva: G. Internal. H. External. I–L Penis: I. Right lateral view. J. Left lateral view. K. Dorsal view. L. Ventral view. Scale bar: A and B = 1 cm. C–L = 1 mm. High quality figures are available online.

***Hypna clytemnestra huebneri* Butler (Figure 10 A–L).** Tegumen, subtraprezoidal in lateral view, anterior projection wider than long, appendices angulares longer than wide. Arms from tegumen and saccus distinct, arms from saccus projected dorsally beyond ventral margin of tegumen. Anterior projection from saccus elongated in lateral view, four times longer than wide and descending; posterior projection absent.

Uncus thick, proximal dorsal portion crestlike, apex slender and descending, in dorsal view not completely fused, uncus with a basal fenestra. Anal tube reduced and entirely membranous. Gnathos open, in lateral view apostrophe-like, proximal portion underneath tegumen, outer distal portion descending and curved, elongate in ventral or posterior views, descending, with distal portion of both gnathos close to each other and enveloped in membranes, membranes joined distally.

Valva approximately rounded. Fultura inferior reduced and V-shaped.

Penis: aedeagus sclerotized, sinuous, with bulbus ejaculatorius projecting from anterodorsal opening, posterior opening sinuous.

**Figure 11.**
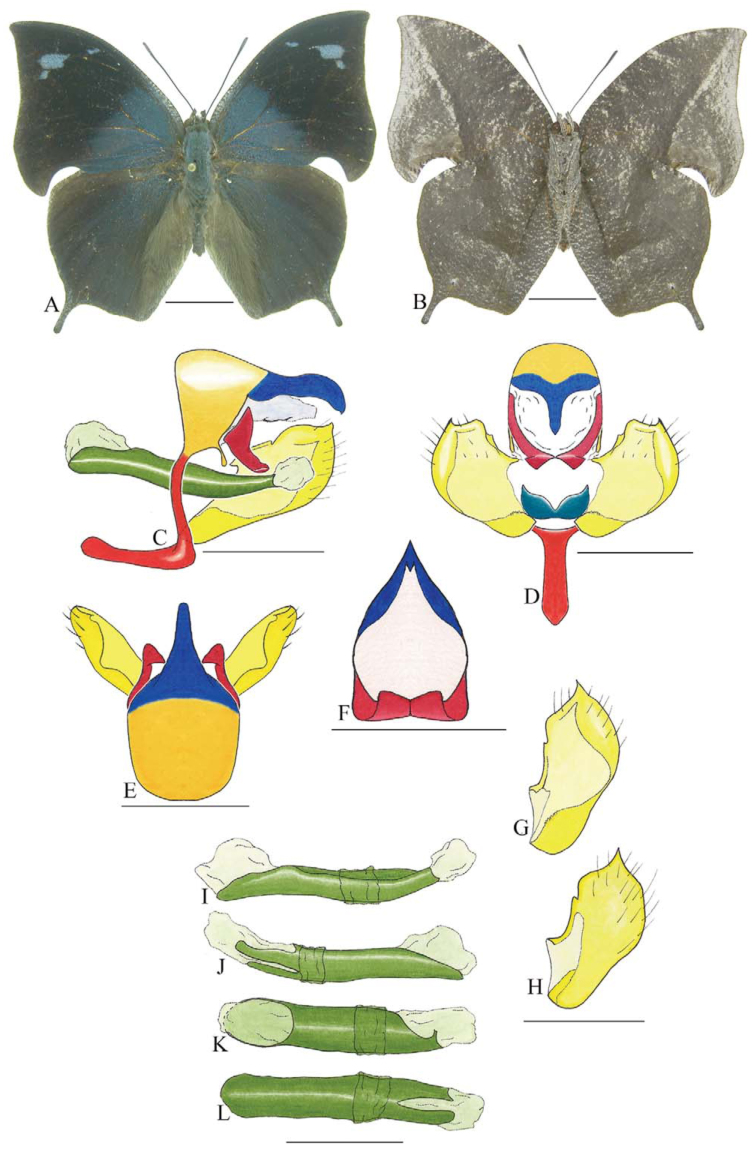
*Memphis acidalia victoria* (H. Druce). A. Dorsal. B. Ventral. C–L Genitalia: C. Lateral view. D. Posterior view. E. Dorsal view. F. Fultura inferior. G and H Valva: G. Internal. H. External. I–L Penis: I. Right lateral view. J. Left lateral view. K. Dorsal view. L. Ventral view. Scale bar: A and B = 1 cm. C–L = 1 mm. High quality figures are available online.

***Memphis acidalia victoria* (H. Druce) (Figure 11 A–L).** Tegumen subtriangular in lateral view, without anterior projection, appendices angulares longer than wide. Arms from tegumen and saccus fused. Anterior projection from saccus elongated in lateral view, distal portion enlarged; posterior projection absent.

Uncus slender, ventral margin sinuous, apex slender and descending, in dorsal view fused with tegumen. Anal tube developed and with ventral portion sclerotized (subscaphium). Gnathos open, in lateral view elongated, with proximal portion wider than long, descending distally and with tegument fold projecting outward like a shell, in ventral view with conspicuous suture indicating the fusion between lateral arms of gnathos.

Valva semi-circular, with reduced spines on sacculus, a small notch on dorsal margin and a spine on distal portion. Fultura inferior bilobed.

Penis: aedeagus sclerotized, with bulbus ejaculatorius projecting from antero-dorsal opening, anterior portion curved descending, and posterior portion with sinuous of different shapes and dimensions.

**Figure 12.**
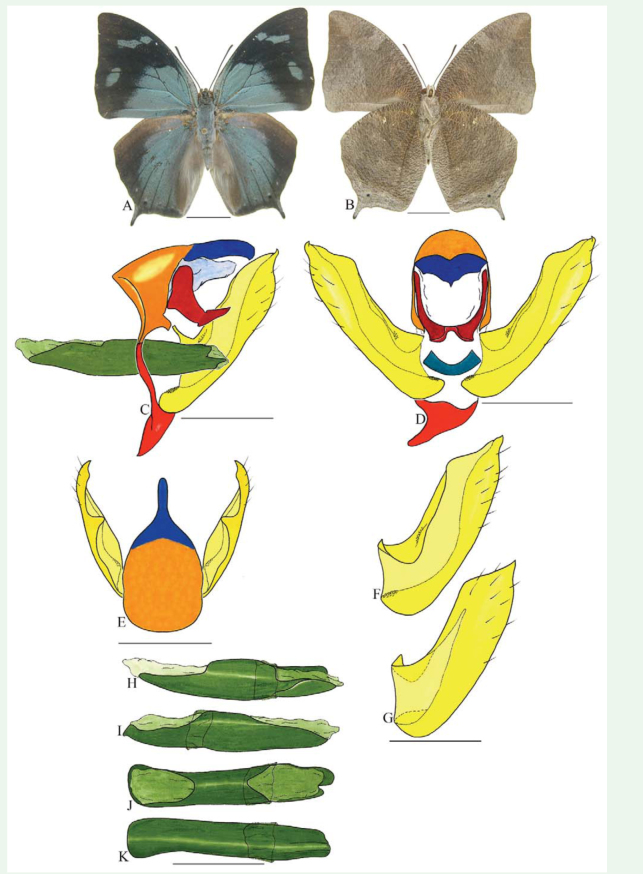
*Memphis glauce glauce* (C. Felder and R. Felder). A. Dorsal. B. Ventral. C–K Genitalia: C. Lateral view. D. Posterior view. E. Dorsal view. F and G Valva: F. Internal. G. External. H–K: Penis: H. Right lateral view. I. Left lateral view. J. Dorsal view. K. Ventral view. Scale bar: A and B = 1 cm. C–K = 1 mm. High quality figures are available online.

***Memphis glauce glauce* (C. Felder and R. Felder) (Figure 12 A–K).** Tegumen subtriangular in lateral view, anterior projection wider than long, tegument fold extended over entire anterior margin, projecting outward, appendices angulares wider than long. Arms from tegumen and saccus distinct. Anterior projection from saccus triangular in lateral view, descending, slender distally, projecting toward the left side; posterior projection present, shorter than anterior projection.

Uncus slender, with ventral margin sinuous, apex descending, in dorsal view fused with tegumen, uncus differing from tegumen by a light sclerotization. Anal tube developed and with ventral portion slcerotized (subscaphium). Gnathos open, in lateral view elongated, proximal portion expanded and distal portion semi-triangular, with median constriction, giving the structure a shovel-like appearance.

Valva subrectangular, each bearing a distal spine; spines reduced on sacculus and costa. Fultura inferior with narrow stripe.

Penis: aedeagus sclerotized, with bulbus ejaculatorius projecting from anterio-dorsal opening, posterior portion with tegument fold near posterior opening.

**Figure 13.**
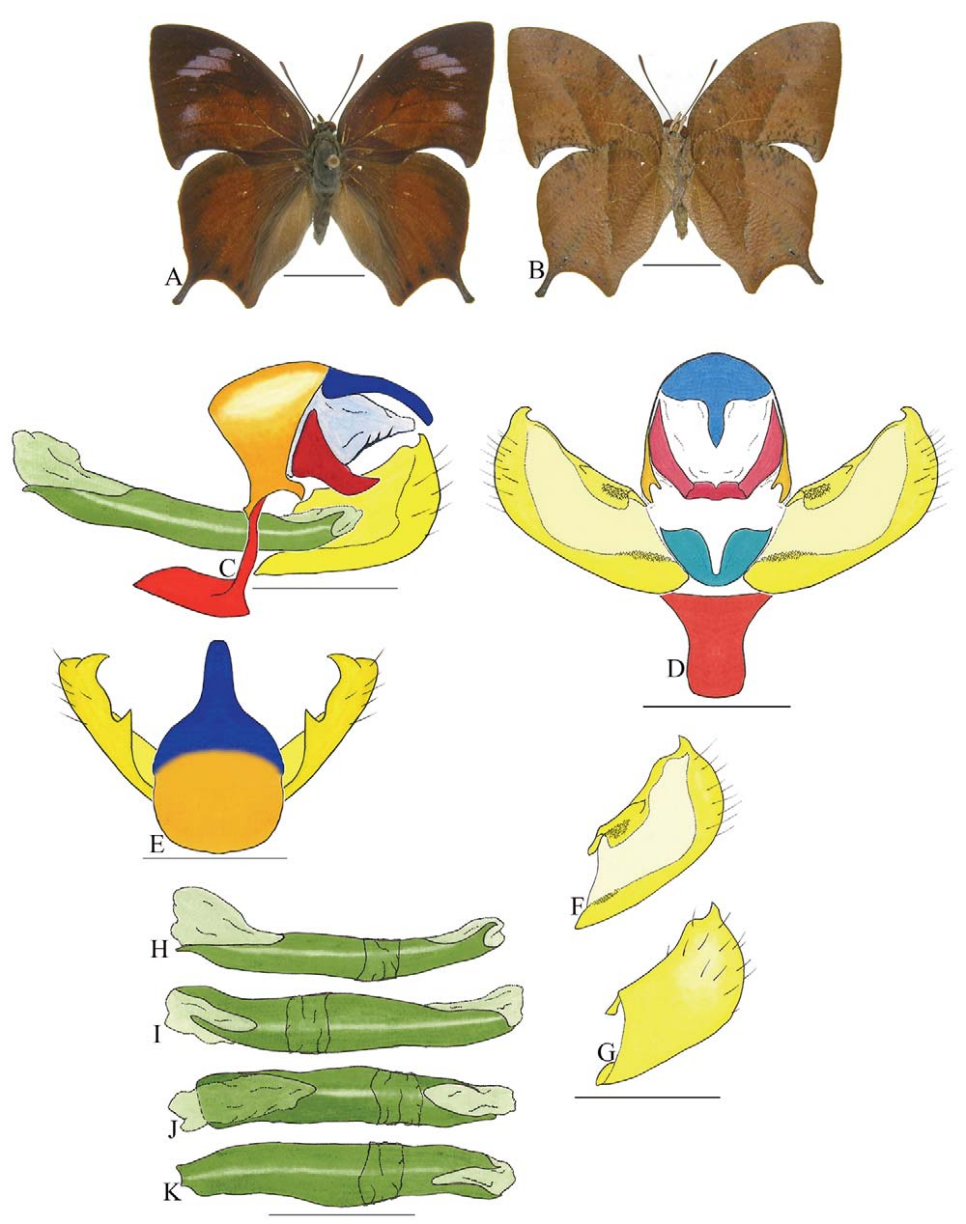
*Memphis hirta* (Weymer). A. Dorsal. B. Ventral. C–K Genitalia: C. Lateral view. D. Posterior view. E. Dorsal view. F and G Valva: F. Internal. G. External. H–K: Penis: H. Right lateral view. I. Left lateral view. J. Dorsal view. K. Ventral view. Scale bar: A and B = 1 cm. C–K = 1 mm. High quality figures are available online.

***Memphis hirta* (Weymer) (Figure 13 A–K).** Tegumen subtriangular in lateral view, anterior projection wider than long, appendices angulares longer than wide. Arms from tegumen and saccus distinct, arm from tegumen with small distal expansions projecting anteriorly. Anterior projection from saccus subrectangular in lateral view, twice as long as wide; posterior projection present, shorter than anterior projection.

Uncus slender, with distal portion descending, in dorsal view fused with tegumen, uncus differing from tegumen by a light esclerotization. Anal tube developed and with ventral portion sclerotized (subscaphium). Gnathos open, in lateral view elongated, proximal portion wider than distal, from posterior view with tegument fold projecting posteriorly on distal portion, shell-like.

Valva semi-circular, each bearing a distal spine, conspicuous spine on dorsal margin and areas with reduced spines on costa and sacculus. Fultura inferior wide and bilobed.

Penis: aedeagus sclerotized, with bulbus ejaculatorius projecting from antero-dorsal opening.

**Figure 14.**
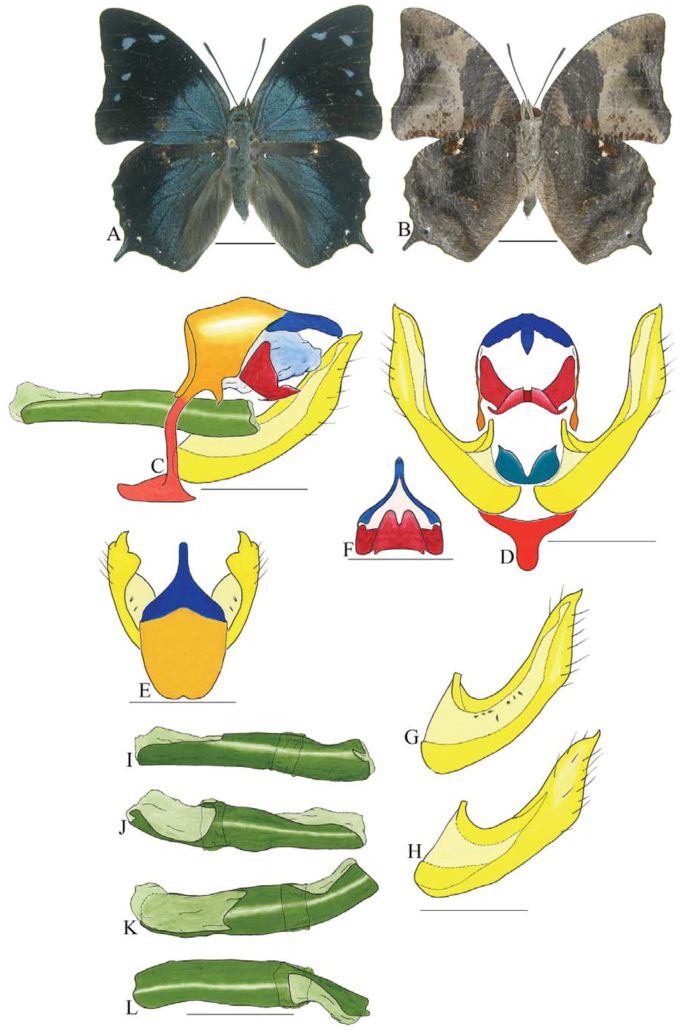
*Memphis lemnos* (H. Druce). A. Dorsal. B. Ventral. C–L Genitalia: C. Lateral view. D. Posterior view. E. Dorsal view. F. Fultura inferior. G and H Valva: G. Internal. H. External. I–L Penis: I. Right lateral view. J. Left lateral view. K. Dorsal view. L. Ventral view. Scale bar: A and B= 1 cm. C–L= 1 mm. High quality figures are available online.

***Memphis lemnos* (H. Druce) (Figure 14 A– L).** Tegumen semi-trapezoidal in lateral view, without anterior projection, tegument fold projecting outward, following anterior margin, appendices angulares longer than wide. Arms from tegumen and saccus fused. Anterior projection from saccus subtriangular in lateral view, slightly longer than wide; posterior projection present, shorter than anterior.

Uncus slender, ventral margin sinuous, apex slender and descending, in dorsal view fused with tegumen, but with proximal lateral portions distinct. Anal tube developed, with ventral portion sclerotized (subscaphium). Gnathos closed, in lateral view elongate, proximal portion wider than distal; distal portion with posterior and anterior projections in posterior view.

Valva subrectangular, bearing distal spine and tufts of setae on inner tegument. Fultura inferior bilobed.

Penis: aedeagus sclerotized, with bulbus ejaculatorius projecting from antero-dorsal opening, posterior-dorsal opening extended to manica.

**Figure 15.**
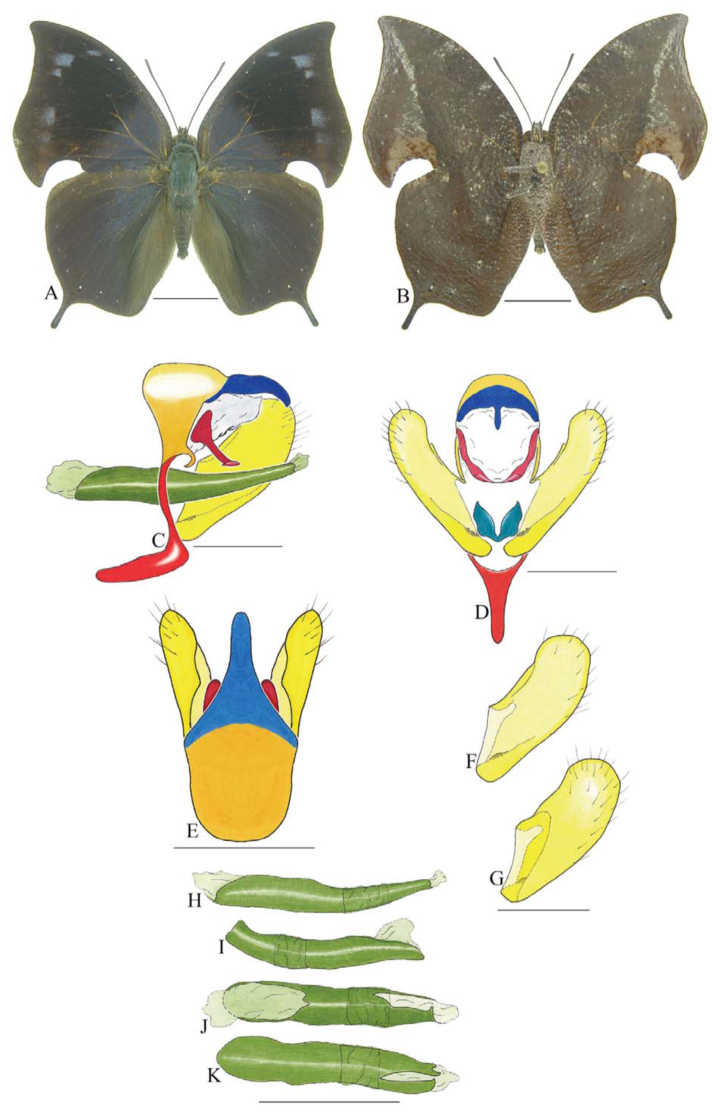
*Memphis moruus stheno* (Prittwitz). A. Dorsal. B. Ventral. C–K Genitalia: C. Lateral view. D. Posterior view. E. Dorsal view. F and G Valva: F. Internal. G. External. H–K: Penis: H. Right lateral view. I. Left lateral view. J. Dorsal view. K. Ventral view. Scale bar: A and B = 1 cm. C–K = 1 mm. High quality figures are available online.

***Memphis moruus stheno* (Prittwitz) (Figure 15 A–K).** Tegumen subtriangular in lateral view, anterior projection wider than long, appendices angulares longer than wide. Arms from tegumen and saccus distinct. Anterior projection from saccus elongated in lateral view, at least three times longer than wide; posterior projection absent.

Uncus slender, ventral margin sinuous and distal portion descending, in dorsal view fused with tegumen, uncus differing from tegumen by a light sclerotization. Anal tube developed, with ventral portion sclerotized (subscaphium). Gnathos closed, in lateral view elongate, proximal portion wider than distal, and with a tegument fold projecting outward.

Valva subrectangular, with small spines on sacculus. Fultura inferior bilobed.

Penis: aedeagus sclerotized, with bulbus ejaculatorius projecting from antero-dorsal opening, posterior-dorsal opening extended to manica.

**Figure 16.**
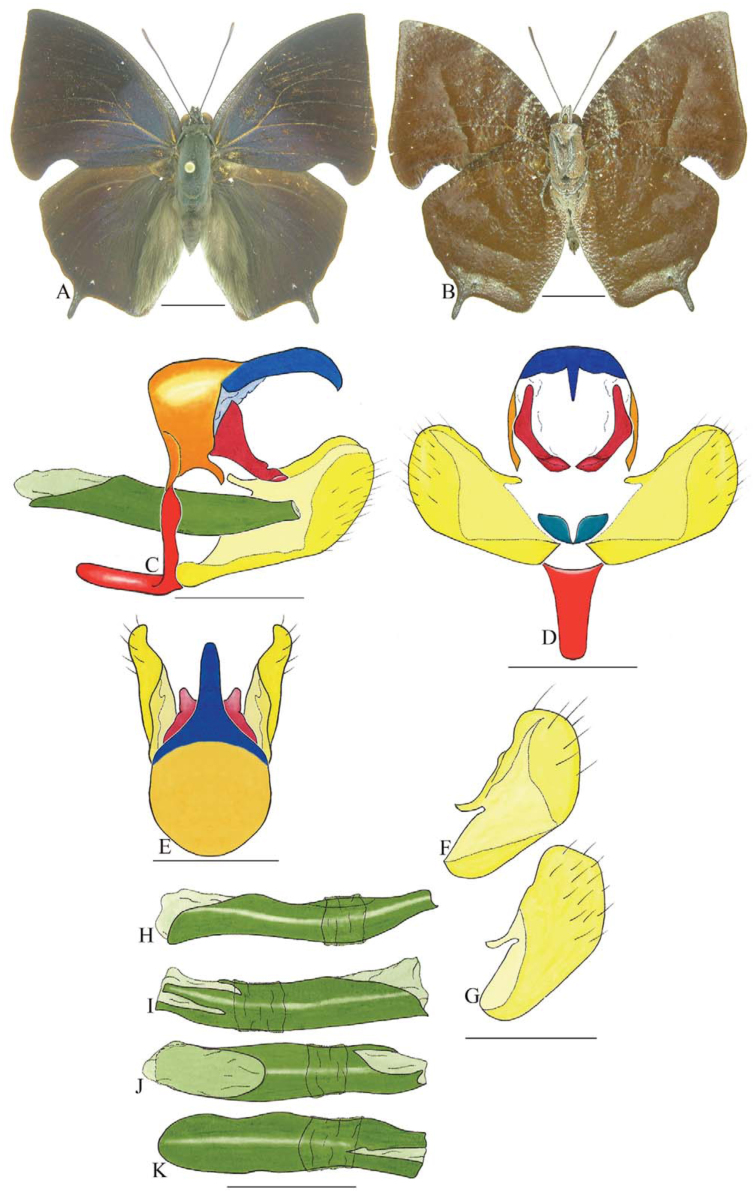
*Memphis philumena corita* (Fruhstorfer). A. Dorsal. B. Ventral. C–K Genitalia: C. Lateral view. D. Posterior view. E. Dorsal view. F and G Valva: F. Internal. G. External. H–K: Penis: H. Right lateral view. I. Left lateral view. J. Dorsal view. K. Ventral view. Scale bar: A and B= 1 cm. C–K= 1 mm. High quality figures are available online.

***Memphis philumena corita* (Fruhstorfer) (Figure 16 A–K).** Tegumen subtrapezoidal in lateral view, anterior projection wider than long, tegument fold projecting outward, appendices angulares longer than wide. Arms from tegumen and saccus distinct. Anterior projection from saccus elongated in lateral view, about four times longer than wide; posterior projection present, shorter than anterior.

Uncus slender, with distal portion descending, in dorsal view fused with tegumen, uncus differing from tegumen by a light sclarotization. Anal tube reduced and entirely membranous. Gnathos open, in lateral view elongate, with proximal portion wider than distal; ventral proximal portion underneath tegumen, with distal tegument fold and dorsal distal notch.

Valva subrectangular and unmodified. Fultura inferior bilobed.

Penis: aedeagus sclerotized, with bulbus ejaculatorius projecting from antero-dorsal opening, posterior opening sinuous.

**Figure 17.**
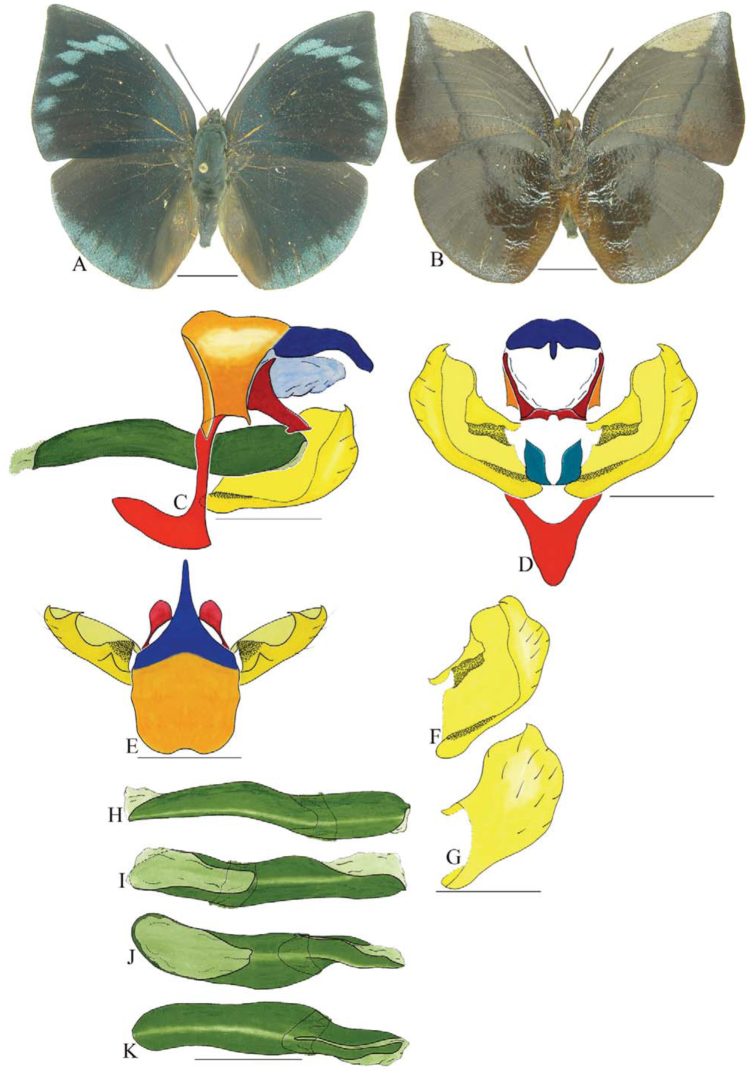
*Memphis polyxo* (H. Druce). A. Dorsal. B. Ventral. C–K Genitalia: C. Lateral view. D. Posterior view. E. Dorsal view. F and G Valva: F. Internal. G. External. H–K: Penis: H. right lateral view. I. Left lateral view. J. Dorsal view. K. Ventral view. Scale bar: A and B= 1 cm. C–K= 1 mm. High quality figures are available online.

***Memphis polyxo* (H. Druce) (Figure 17 A– K).** Tegumen subtrapezoidal in lateral view, anterior projection wider than long, tegument fold on anterior margin, projected outward, appendices angulares longer than wide. Arms from tegumen and saccus distinct. Anterior projection from saccus elongated in lateral view, twice as long as wide; posterior projection absent.

Uncus slender, with proximal portion enlarged and distal portion descending, in dorsal view fused with tegumen, uncus differing from tegumen by a light sclerotization. Anal tube developed, with ventral portion sclerotized (subscaphium). Gnathos closed, in lateral view elongate, proximal portion about twice wider than distal portion, tegument fold projecting outward.

Valva subrectangular, each bearing a distal spine, spines on sacculus and inner tegument small, near costa. Fultura inferior bilobed.

Penis: aedeagus sclerotized and sinuous, with bulbus ejaculatorius projecting from anterodorsal opening, posterior portion with tegument fold, posterior opening sinuous.

**Figure 18.**
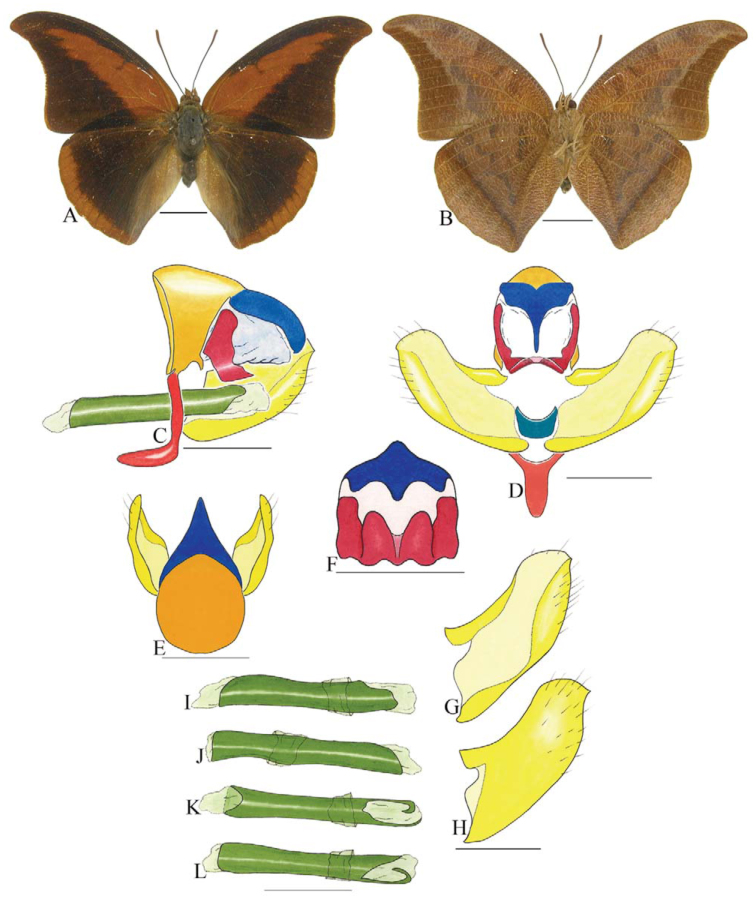
*Prozikania suprema* (Schaus). A. Dorsal. B. Ventral. C–L Genitalia: C. Lateral view. D. Posterior view. E. Dorsal view. F. Fultura inferior. G and H Valva: G. Internal. H. External. I–L Penis: I. Right lateral view. J. Left lateral view. K. Dorsal view. L. Ventral view. Scale bar: A and B = 1 cm. C–L = 1 mm. High quality figures are available online.

***Prozikania suprema* (Schaus) (Figure 18 A– L).** Tegumen subtriangular in lateral view, without anterior projection, tegument fold projected outwards on anterior margin, appendices angulares longer than wide. Arms from tegumen and saccus fused. Anterior projection from saccus elongated in lateral view, at least twice longer than wide; posterior projection absent.

Uncus slender, apex slender and descending, in dorsal view fused with tegumen, uncus differing from tegumen by a light sclerotization. Anal tube developed, with ventral portion sclerotized (subscaphium). Gnathos closed, in lateral view elongate, proximal portion twice as wide as distal, outer margin sinuous, distal tegument fold projecting outward.

Valva subrectangular, distally with outer tegument expanded. Fultura inferior wide and forming a semi-circle.

Penis: aedeagus sclerotized, with bulbus ejaculatorius projecting from antero-dorsal opening, posterior opening irregular.

**Figure 19.**
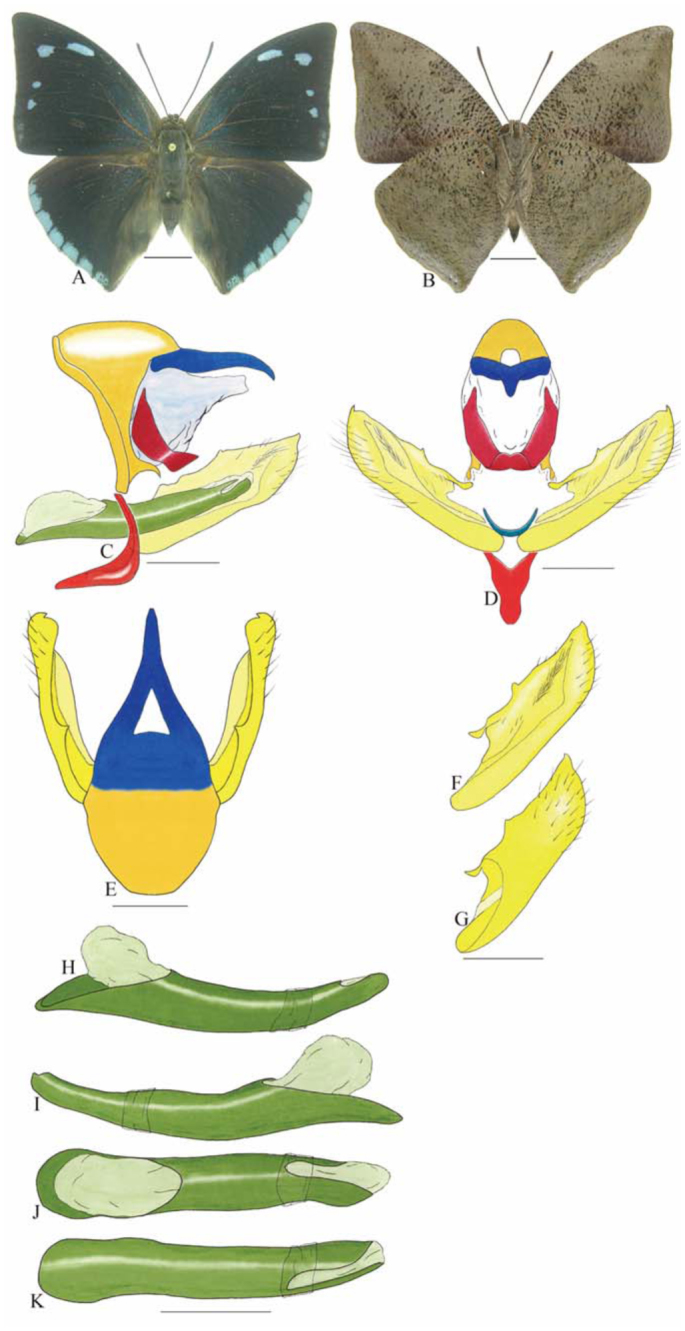
*Pseudocharaxes xenocrates punctimarginale* (Kaye). A. Dorsal. B. Ventral. C–K Genitalia: C. lateral view. D. posterior view. E. Dorsal view. F and G Valva: F. Internal. G. External. H–K: Penis: H. right lateral view. I. left lateral view. J. dorsal view. K. ventral view. Scale bar: A and B = 1 cm. C–K = 1 mm. High quality figures are available online.

***Pseudocharaxes xenocrates punctimarginale* (Kaye) (Figure 19 A–K).** Tegumen subtriangular in lateral view, anterior projection wider than long, tegument fold of anterior margin projected outward, appendices angulares longer than wide. Arms from tegumen and saccus separated, arms from tegumen long, the longest among the species studied in this work. Anterior projection fom saccus semi-triangular in lateral view, about twice as long as wide; posterior projection absent.

Uncus slender, ventral margin sinuous, apex slender and descending, in dorsal view fused with tegument, with triangular fenestra medially. Anal tube developed, with ventral portion sclerotized (subscaphium). Gnathos closed, in lateral view elongate, with distal tegument fold projected outward, giving the ghathos a shell-like appearance.

Valva rectangular, dorsal margin sinuous, spine on distal portion and setae on inner region, setae more numerous than in other species analyzed. Fultura inferior small and semi-circular.

Penis: aedeagus sclerotized, with bulbus ejaculatorius projecting from antero-dorsal opening, posterior opening sinuous.

**Figure 20.**
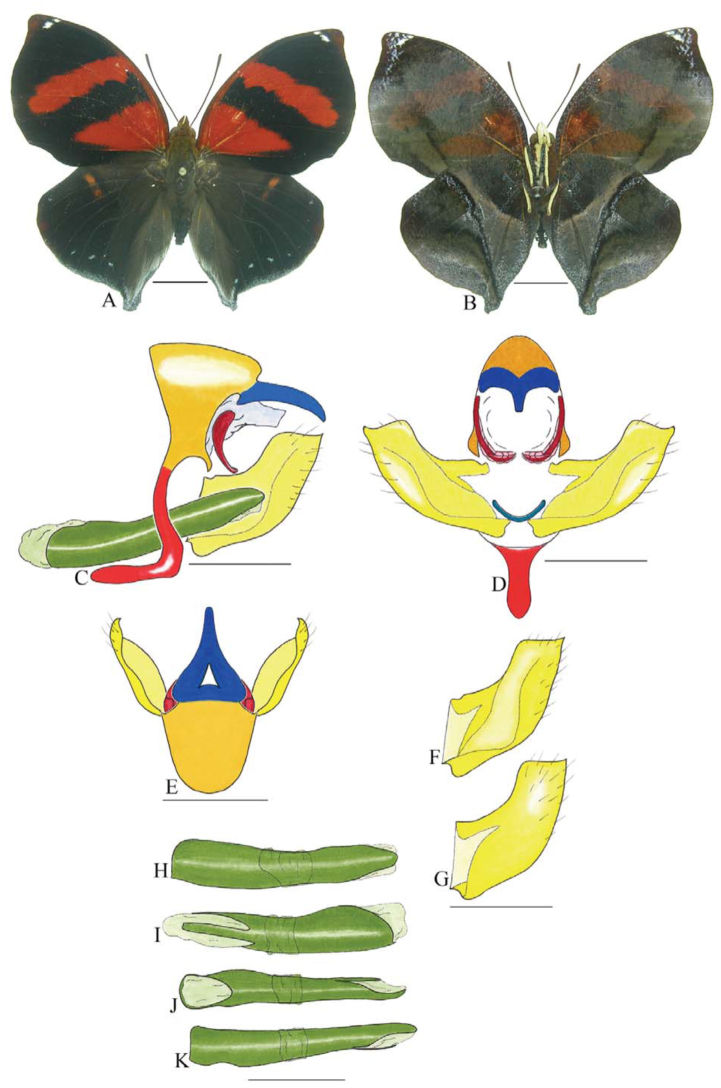
*Siderone nemesis catarina* Dottax and Pierre. A. Dorsal. B. Ventral. C–K Genitalia: C. Lateral view. D. Posterior view. E. Dorsal view. F and G Valva: F. Internal. G. External. H–K: Penis: H. Right lateral view. I. Left lateral view. J. Dorsal view. K. Ventral view. Scale bar: A and B = 1 cm. C–K = 1 mm. High quality figures are available online.

***Siderone nemesis catarina* Dottax and Pierre (Figure 20 A–K).** Tegumen subtriangular in lateral view, anterior projection wider than long, appendices angulares longer than wide. Arms from tegumen and saccus fused. Anterior projection from saccus elongated in lateral view, at least four times longer than wide; posterior projection absent.

Uncus slender, with distal portion descending, in dorsal view fused with tegumen, with triangular fenestra medially. Anal tube developed, with ventral portion sclerotized (subscaphium). Gnathos open, in lateral view elongate, completely enveloped by membranes.

Valva rectangular. Fultura inferior narrow and semi-circular.

Penis: aedeagus sclerotized, with bulbus ejaculatorius projecting from antero-dorsal opening, posterior opening large and sinuous.

**Figure 21.**
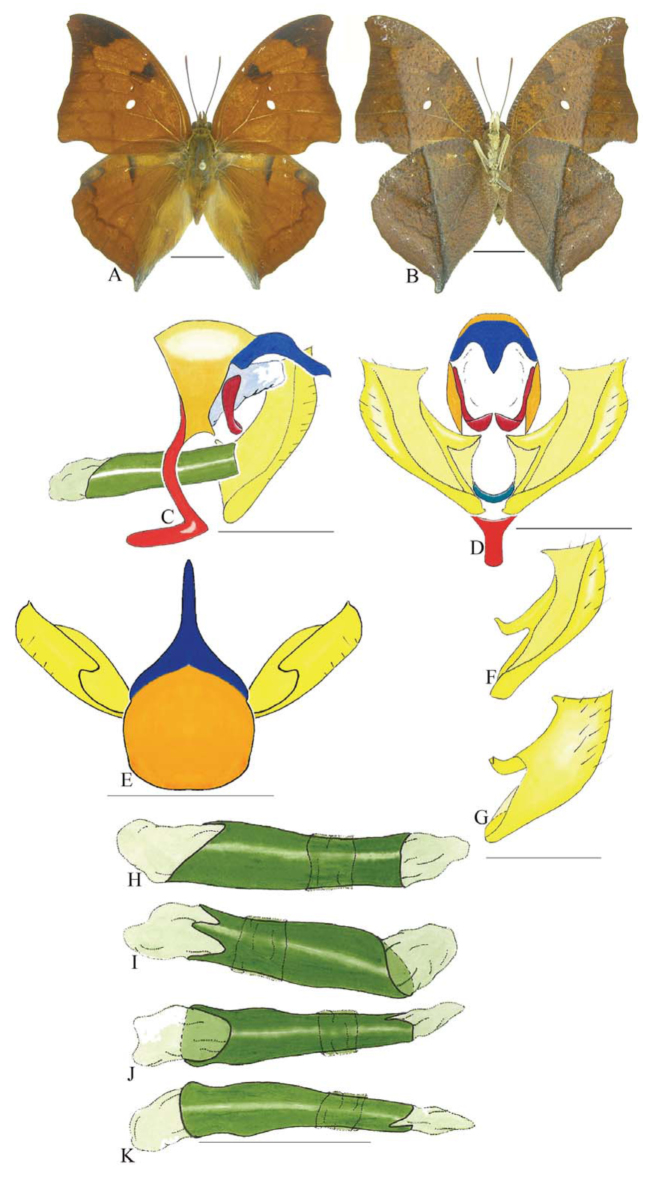
*Zaretis isidora* (Cramer). A. Dorsal. B. Ventral. C–K Genitalia: C. lateral view. D. Posterior view. E. Dorsal view. F and G Valva: F. Internal. G. External. H–K: Penis: H. Right lateral view. I. Left lateral view. J. Dorsal view. K. Ventral view. Scale bar: A and B = 1 cm. C–K = 1 mm. High quality figures are available online.

***Zaretis isidora* (Cramer) (Figure 21 A–K).** Tegumen subtriangular in lateral view, anterior projection wider than long, appendices angulares as wide as long. Arms from tegumen and saccus distinct. Anterior projection from saccus elongated in lateral view, at least three times longer than wide; posterior projection from saccus absent.

Uncus slender, apex slender and descending, in dorsal view fused with tegumen, distinct from tegumen by light sclerotization. Anal tube developed, with ventral portion sclerotized (subscaphium). Gnathos open, in lateral view elongate, distal portion of both gnathos close to each other, tegument fold on distal portion shell-like.

Valva rectangular, with inner and outer ends conspicuous. Fultura inferior narrow and semi-circular.

Penis: aedeagus sclerotized, with bulbus ejaculatorius projecting from antero-dorsal opening.

### Anaeomorphini

**Figure 22.**
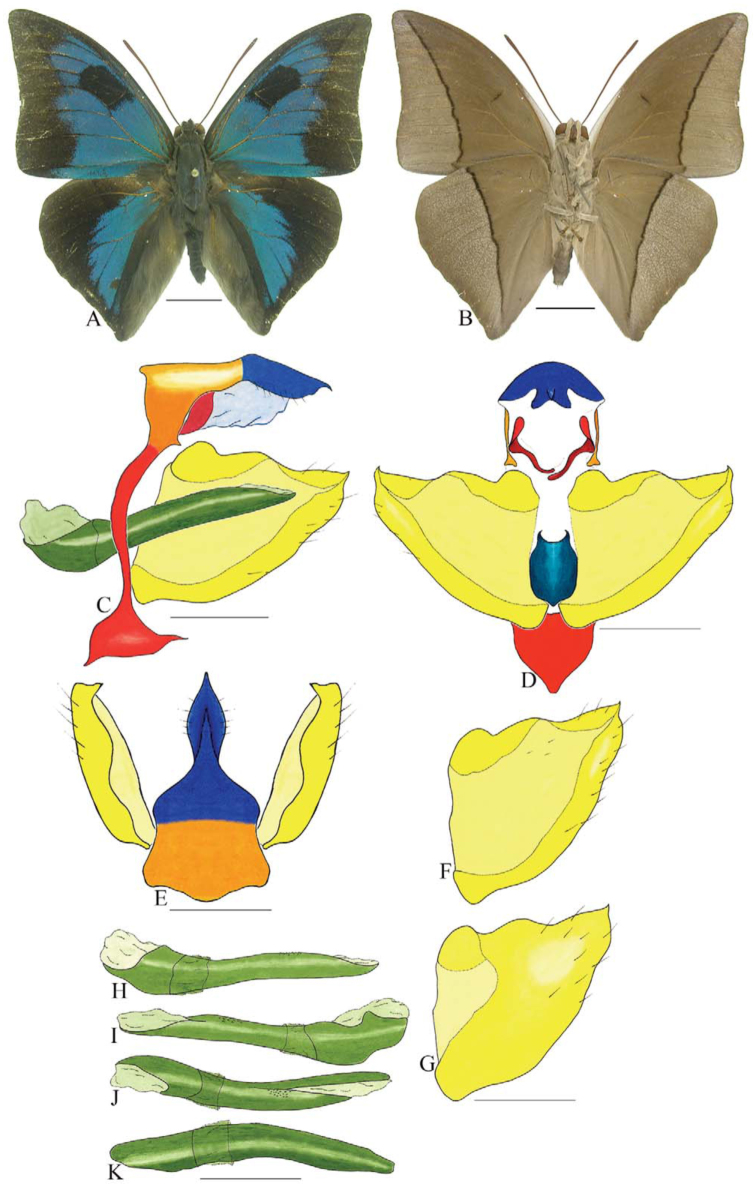
*Anaeomorpha splendida* Rothschild. A. Dorsal. B. Ventral. C–K Genitalia: C. Lateral view. D. Posterior view. E. Dorsal view. F and G Valva: F. Internal. G. External. H–K: Penis: H. Right lateral view. I. Left lateral view. J. Dorsal view. K. Ventral view. Scale bar: A and B = 1 cm. C–K = 1 mm. High quality figures are available online.

***Anaeomorpha splendida* Rothschild (Figure 22 A–K).** Tegumen triangular in lateral view, anterior projection as wide as long, slightly concave on distal portion; appendices angulares wider than long. Arms from tegumen and saccus fused. Anterior projection from saccus subtriangular, in lateral view, wider than long; posterior projection present, subequal to anterior projection, with sharp tip.

Uncus thick, as large as tegumen, apex slender and descending, in dorsal view fused with tegumen, uncus differing from tegumen by a light sclerotization, setae on ventral margin at the beginning of tegument fold, which projects latero-ventrally and has a row of spines near distal portion. Anal tube developed and entirely membranous. Gnathos open, in lateral view with proximal and distal portions covered by the tegumen, in posterior view L-shaped, distal portion longer than proximal.

Valva subtriangular, sharp on distal portion. Fultura inferior rectangular, twice as long as tall, with concave dorsal margin.

Penis: aedeagus sclerotized, with bulbus ejaculatorius projecting from antero-dorsal opening. This is the only species studied with small spines on dorsal portion, near the source of the posterior opening.

### Preponini

***Archaeoprepona amphimachus pseudomeander* (Fruhstorfer) (Figure 24 A–L, 33 E).** Tegumen rectangular, anterior projection as wide as long, anterior tegument fold projecting outward; appendices angulares wider than long. Arms from tegumen and saccus fused. Anterior projection from saccus subrectangular in lateral view; posterior projection present, shorter than anterior.

Uncus thick, with short setae on ventral distal portion, apex slender, in dorsal view fused with tegumen, uncus differing from tegumen by a light sclerotization. Anal tube reduced and entirely membranous. Gnathos open, in lateral view long, with descending projection, apex shaped like a club, concave, striated.

Valva semi-triangular, distal expansion of inner tegument with short setae, conspicuous spine arising from outer tegument. Fultura inferior bilobed with dorsal median notch.

Penis: aedeagus membranous ventrally, with bulbus ejaculatorius projecting from antero-drosal opening.

***Archaeoprepona chromus chromus* (Guérin-Méneville) (Figure 24 A–K).** Tegumen rectangular in lateral view, anterior projection wider than long, fold from anterior tegument projecting outward, appendices angulares longer than wide. Arms from tegumen and saccus distinct. Anterior projection from saccus elongated dorsoventrally in lateral view; posterior projection shorter than anterior projection, with sharp apex.

Uncus thick, in dorsal view fused with tegumen, uncus differing from tegumen by a light sclerotization, dorsal lobe with conspicuous boundaries. Anal tube reduced and entirely membranous. Gnathos open, in lateral view with proximal portion elongated, distal portion descending and striated.

Valva semi-triangular, distal expansion of tegument, and distal spine. Fultura inferior bilobed, with dorsal and ventral notches.

Penis: aedeagus with ventral portion membranous, bulbus ejaculatorius projecting from antero-ventral opening.

***Archaeoprepona demophon muson*(Fruhstorfer) (Figure 25 A–K).** Tegumen rectangular in lateral view, anterior projection as wide as long, anterior tegument fold projected outward, appendices angulares wider than long. Arms from tegumen and saccus fused. Anterior projection from saccus absent; posterior projection present, developed, subtriangular with distal portion sharp.

Uncus thick, in dorsal view fused with tegumen, uncus differing from tegumen by a light sclerotization, dorsal lobe with conspicuous boundaries and enveloped by numerous spines on lateral portion. Anal tube reduced and entirely membranous. Gnathos open, in lateral view comma-like, striated on medial distal portion.

Valva subtriangular, distal expansions of inner tegument, with spine arising from outer tegument and with long setae. Fultura inferior bilobed and articulated with ventral area of valva.

Penis: aedeagus, ventral portion membranous, medial-anterior region dilated in lateral view, narrow in dorsal or ventral views, bulbus ejaculatorius projecting from antero-dorsal opening.

***Archaeoprepona demophoon andicola* (Fruhstorfer) (Figure 26 A–G, 27 A–E).** Tegumen rectangular in lateral view, anterior projection wider than long, tegument fold reduced and covering part of the hollow, which distinguishes the arms from tegumen; appendices angulares wider than long. Arms from tegumen and saccus distinct. Anterior projection from saccus subrectangular in lateral view, twice as long as wide; posterior projection present shorter than anterior projection, slender distally and descending.

Uncus thick, in dorsal view fused with tegumen, uncus differing from tegumen by a light sclerotization, dorsal lobe with conspicuous boundaries. Anal tube reduced and entirely membranous. Gnathos open, in lateral view comma-like, striated on distal two thirds.

Valva semi-triangular, distal expansion of inner tegument, spine arising from outer tegument. Fultura inferior bilobed and dorsal inner margins rounded.

Penis: aedeagus wider dorsoventrally when compared with other species of the genus, anterior ventral portion rounded, small anterodorsal lobe and median constriction preceding manica, ventral portion membranous, bulbus ejaculatorius projecting from antero-dorsal opening.

***Archaeoprepona licomedes licomedes* (Cramer) (Figure 28 A–L).** Tegumen rectangular in lateral view, anterior projection wider than long, anterior ventral fold projected outward, appendices angulares wider than long, ventral margin sinuous. Arms from tegumen and saccus distinct. Anterior projection from saccus subtriangular in lateral view; posterior projection shorter than anterior, diamond-shaped.

Uncus thick, in dorsal view fused with tegument, uncus differing from tegumen by a light sclerotization, dorsal lobe with conspicuous boundaries. Anal tube reduced and entirely membranous. Gnathos open, in lateral view with two flattened, folded plates in lateral view, distal plate descending and striated on outer portion.

Valva subtriangular, distal expansion of inner tegument, and distal spine arising from outer tegument. Fultura inferior bilobed, lateral margins sinuous, a smooth ventral-median notch and deep dorsal median notch.

Penis: aedeagus, ventral portion entirely membranous, bulbus ejaculatorius projecting apically

***Archaeoprepona meander castorina* (E. Mayr) (Figure 29 A–K).** Tegumen rectangular in lateral view, anterior projection wider than long, tegument fold small and projected outward, appendices angulares longer than wide. Arms from tegumen and saccus distinct. Anterior projection of saccus semi-triangular in lateral view, longer than wide; posterior projection present, shorter than anterior projection.

Uncus slender, with expansion from median to distal portion, ventral margin sinuous, apex slender and descending, in dorsal view fused with tegumen, uncus differing from tegumen by a light sclerotization. Anal tube reduced and entirely membranous. Gnathos open with distal portion striated.

Valva semi-triangular, distal expansion arising from inner tegument, and distal spine arising from outer tegument. Fultura inferior bilobed.

Penis: aedeagus with a concavity on ventral median portion, membranous ventrally, bulbus ejaculatorius projecting apically.

***Prepona claudina annetta* (Gray) (Figure 30 A–K).** Tegumen semi-trapezoidal in lateral view, anterior projection wider than long, deep depression on posterior ventral portion, appendices angulares wider than long. Arms from tegumen and saccus distinct. Anterior projection from saccus oval in lateral view, longer than wide; posterior projection present, shorter than anterior.

Uncus thick, elevated on proximal dorsal portion, with distal portion descending, in dorsal view fused with tegumen, uncus differing from tegumen by a light sclarotization. Anal tube reduced, entirely membranous and linked to the gnathos. Gnathos open, in lateral view elongate, with long arm and with spiny apical club on, projected dorsally.

Valva triangular. Fultura inferior rectangular, taller than wide, dorsally with two convex areas covered with small spines, tegument fold projecting laterally.

Penis: aedeagus sclerotized dorsally and with ventral sclerotization restricted to median region, bulbus ejaculatorius projects from antero-ventral opening.

***Prepona laertes laertes* (Hubner) (Figure 31 A–K).** Tegumen semi-trapezoidal in lateral view, anterior projection wider than long, dorsal margin with small folds, appendices angulares wider than long. Arms from tegumen and saccus distinct. Anterior projection from saccus oval in lateral view; posterior projection absent.

Uncus thick, apex slender and descending, in dorsal view fused with tegumen, conspicuous constriction between uncus and tegumen. Anal tube reduced and entirely membranous. Gnathos open, in lateral view long, with spiny club projected dorsally, and extended beyond uncus.

Valva triangular. Fultura inferior rectangular, taller than wide, with basal lateral folds projected outward.

Penis: aedeagus sclerotized, with tegument fold preceding manica, bulbus ejaculatorius projecting from antero-ventral opening.

***Prepona pylene pylene* Hewitson (Figure 32 A–K).** Tegumen semi-trapezoidal, anterior projection wider than long, appendices angulares wider than long. Arms from tegumen and saccus distinct. Anterior projection from saccus oval in lateral view; posterior projection absent.

Uncus thick, with distal portion concave and descending, in dorsal view fused with tegumen, uncus differing from tegumen by the latter through a dorsal process on the proximal portion. Anal tube reduced and entirely membranous. Gnathos open, in lateral view long, with spiny club projecting dorsally, and extended beyond uncus.

Valva triangular. Fultura inferior taller than wide, with lateral fold projecting outward.

Penis: aedeagus sclerotized, bulbus ejaculatorius projecting from antero-ventral opening.

## Discussion

### Tegumen

This sclerite is structurally diverse across the group, with differences in at least five areas. Four shapes have been identified in lateral view: (1) semi-trapezoidal, clustering species of *Agrias, Prepona,* and *Hypna,* as well as *Memphis philumena corita, Memphis lemnos,* and *Memphis polyxo*; (2) rectangular, common to species of *Archaeoprepona* and *Noreppa*; (3) triangular, clustering *Anaea troglodyta* with *Anaeomorpha splendida*; and (4) subtriangular, common among species of Anaeini, and clustering species of *Coenophlebia, Consul, Fountainea, Prozikania, Pseudocharaxes, Siderone, Zaretis,* and most species *of Memphis.*

With a few exceptions, enumerated below, the anterior projection of the tegumen is developed. It is lacking in most species of *Fountainea* (the anterior projection is found only in *Fountainea ryphea phidile,* of the four species studied) and a few species of Anaeini (*Memphis acidalia victoria, Memphis lemnos,* and *Prozikania suprema*). When present, the anterior projection of the tegumen is often developed, longer than wide or as wide as long. Such projection is reduced or is as wide as long only in *Anaeomorpha splendida, Archaeoprepona* amphimachus *pseudomeander,* and *Archaeoprepna demophon muson.*

Other modifications in the tegumen were the presence or absence of a fold in the tegument on the anterior margin and appendices angulares. The latter are ubiquitous among the species studied and vary in their dimensions, being wider than long, as wide as long, or longer than wide.

### Saccus

Some differentiation between the ventral arms from the tegumen and the dorsal arms from the saccus was found in 23 of the 31 species analyzed, with special emphasis for *P. xenocrates punctimarginale*, with the arms of the tegumen separated and distant from the arms of the saccus. Therefore, the fusion between these two structures has not revealed a suitable character to group species or genera.

Thirty species have a saccus with an anterior projection. The degree of development of this projection varies among species, and six shapes have been identified for it: oval, common among species of *Agrias* and *Prepona;* rectangular, a shape found only in *Anaea troglodyta;* subtriangular, characteristic of *Anaeomorpha splendida, Archaeoprepona demophoon andicola, Archaeoprepona licomedes licomedes, Pseudocharaxes xenocrates punctimarginale,* and *Memphis lemnos;* subrectangular, as in *Archaeoprepona amphimachus* pseudomeander, *Archaeoprepona domophon muson,* and *Memphis hirta;* triangular, as in *Archaeoprepona meander castorina* and *Memphis glauce glauce;* and elongated, characteristic of species of *Consul, Fountainea* and *Hypna,* as well as *Coenophlebia archidona, Noreppa chromus chromus, Prozikania suprema, Siderone nemesis catarina, Zaretis isidora, Memphis acidalia victoria, Memphis moruus stheno, Memphis phlilumena corita,* and *Memphis polyxo.*

A posterior projection arising from the saccus was present in approximately 50% of the species studied. This projection can be shorter than the anterior projection, a widespread state, or it can be subequal to or longer than the anterior projection, a state present in *Anaeomorpha splendida* and *Archaeoprepona domophon muson.*

### Uncus

Among the structural differences found in the uncus, we highlight its shape in lateral view, which can be thick (three times longer than wide) or slender (more than three times longer than wide). The tribe *Preponini* contains only one species with a slender uncus, *Archaeoprepona meander meander.* All other species in this tribe, as well as the species *Hypna* and *Coenophlebia* (Anaeini), have a thick uncus. The uncus is slender in species of *Anaea, Consul, Memphis, Fountainea, Siderone, Prozikania, Pseudocharaxes,* and *Zaretis.*

The uncus also varies in the presence or absence of setae on its dorsal portion. The uncus of *Anaeomorpha splendida, Archaeoprepona* amphimachus *pseudomeander,* and *Prepona laertes laertes* have a dorsal seta. Other differences in this structure among species are as follows: the presence or absence of crests (present only in two subspecies of *Hypna clytemnestra*), presence or absence of spines on the dorsal portion (present in *Anaeomorpha splendida, Archaeoprepona* amphimachus *pseudomeander,* and *Prepona laertes laerte*), and the presence or absence of a fenestra. When present, the fenestra may be found either on the central portion (*Pseudocharaxes xenocrates punctimarginale* and *Siderone nemesis catarina*) or at the base of the uncus (*Hypna clytemnestra forbesi* and *Hypna clytemnestra huebneri*).

### Anal tube

The degree of sclerotization of the ventral portion was the only difference found in the anal tube. Species that have the ventral portion of the anal tube sclerotized were considered as having a subscaphium. This character can be used to separate between Anaeini, Anaeomorphini and Preponini. Species of Preponini and Anaeomorphini do not have a subscaphium, whereas species of Anaeini do, with two exceptions, *Fountainea nessus* and *Fountainea ryphea phidile.*

### Gnathos

The gnathos has two basic shapes in the species studied. It may form a handle enveloping the anal tube, in which case it is called “single” or “close,” or it may be divided into two distinct arms, and be called “double” or “open.” In our sample, the gnathos is single in species of Anaeini and double in species of and Anaeomorphini Preponini, with some modifications among species.

Five aspects were identified concerning the shape of the gnathos: elongated, present in species of *Agrias* and *Prepona*; shaped as an anvil, unique to *Anaea troglodyta*; covered by the tegument, unique to *Anaeomorpha splendida;* thick, present in *Archaeoprepona* and *Noreppa;* with base wider than distal portion, as in Anaeini, except for *Anaea troglodyta.* Regarding the modifications on the distal portion, they can be smooth, with spiny club, or with distal margin serrated or striated and enveloped by membranes.

### Valvae

The valvae assume various shapes: triangular, as in *Agrias* and *Prepona;* subtriangular, in species of *Anaea, Anaeomorpha,* and *Archaeoprepona;* circular, common to the species of *Hypna* and *Consul,* as well as *Memphis acidalia victoria, Memphis hirta,* and *Memhis polyxo;* rectangular, as in *Pseudocharaxes xenocrates punctimarginale,* *Siderone nemesis catarina,* and *Zaretis isidora;* and subrectangular, as in all species of *Fountainea, Coenophlebia archidona, Memphis philumena corita, Memphis moruus stheno, Memphis lemnos, Memphis glauce glauce,* and *Prozikania suprema.*

Besides the shape, the structure of the valvae may also vary. For instance, the presence of an apical projection is a variable feature within tribes, but stable within genera. In our sample, the valva of all species of *Archaeoprepona, Fountainea, Consul, Prozikania, Pseudocharaxes,* and *Memphis* (except for *Memphis moruus stheno*) had an apical projection.

Beyond the characters described above, the following features have been found to be variable: presence or absence of spines on costa (present in *Fountainea halice halice, Memphis glauce glauce, Memphis hirta, Memphis polyxo*); presence or absence of spines on the saccus (present in species of *Memphis*); presence or absence of an apical projection of the valva, presence or absence of setae on the inner tegument of the valva (present only in *Memphis lemnos* and *Pseudocharaxes xenocrates punctimarginale*). These characters can be further investigated, and the location and shape of spines can be detailed.

### Fultura inferior

Differences were found in the fultura inferior of all species. Within this structural diversity, we were able to devise some basic aspects. One is the general shape, with six variations: rectangular, as in all species of *Agrias* and *Prepona;* semi-circular, as in *Anaeomorpha splendida, Fountainea ryphea phidile, Prozikania suprema,* and *Pseudocharaxes xenocrates punctimarginale;* elongated, present only in *Coenophlebia archidona;* V-shaped, present among *Hypna* subspecies; narrow, stripe-like, unique to *Memphis hirta;* and bilobed, present in all other species analyzed.

Other characters identified for the fultura inferior were useful for grouping species, such as the presence of absence of tegument folds, uniting species of *Agrias* and *Prepona.* Another such character is the presence or absence of spines and their location.

### Penis

The penis presents variations in the aedeagus, which can be completely sclerotized or membranous ventrally. A ventrally membranous aedeagus was found in the studied species of *Archaeoprepona* and *Noreppa.*

Another character that varied in the penis was the direction of the projection from the bulbus ejaculatorius. It was ventral in species of *Agrias* and *Prepona,* and also in *Noreppa chromus chromus.* In *Archaeoprepona licomedes licomedes* and *Archaeoprepona meander castorina,* this projection was ventral. It was dorsal in 25 of the 31 species studied.

### Final considerations

The present analysis revealed the importance of conducting detailed morphological analyses that focus on certain tagmas, particularly for groups whose morphology has been neglected, such as the Charaxinae. It is possible that this group has been poorly studied because specimens are rare and consequently they are considered precious. For this reason, their taxonomy has been based on venation and coloration of the wings only.

The observation of Rydon (1971) was confirmed, because the differences of the gnathos, club-like and striated-like, were able to differentiate *Archaeoprepona* and *Prepona.* According to the results, species of Preponini can be divided into two distinct groups, *Prepona* (including *Prepona claudina annetta*) and *Archaeoprepona* (including *Archaeoprepona chromus chromus*), thus confirming the arrangement of the genera proposed by Ortiz-Acevedo and Willmott (2013).

Among the characters observed in this study, the following putative synapomorphies support groups within Preponini:

*Archaeoprepona:* Aedeagus with ventral portion membranous and distal portion of gnathos striated;

*Prepona:* Presence of lateral folds in the tegument of the fultura inferior, and gnathos with a club covered with spines.

Morphological characters can resolve taxonomic problems, and can still be more exploited, thus making them more accurate when included in analyses such as female genitalia, color and shape of wings, and even the shape and color of odoriferous scales in males.

The structural modifications of *Anaeomorpha splendida* confirm a tribe, although in some aspects it resembles *Anaea troglodyta,* which deserves attention and further research about the degree of closeness between these two species belonging to different tribes, Anaeomorphini and Anaeini, respectively.

Contrasting with the genitalia of species of Preponini, the gentialia of Anaeini species is variable, making it difficult to establish structural patterns within this group. However, it is possible to distinguish among species based on some genitalic characters. For instance, species of *Memphis* and *Fountainea* are easily separated by the presence and location of spines on the valva. For Comstock (1961), the great variety of forms of gnathos, which despite having a structural pattern as a basal line can provide many features useful for the determination of species.

The results obtained herein reflect detailed dissections and observations of structures from different views (positions) that had not been previously used in the taxonomy of the group. Therefore, they include structures not previously considered. These characters can be used in the future in a phylogenetic analysis or when devising more consistent diagnostic characters for species and genera.

The detailing of such structures can be geared towards the maintenance of the status of *Prozikania* and *Pseudocharaxes* as genera, synonymized by Lamas (2004) with *Polygrapha,* the first as *Zikania* (nom. nud.), and again used by Salazar (2008). The significant differences in the genitalia of the species of both genera as described and illustrated in this study corroborate the observations of Comstock (1961), Rydon (1971), and Salazar (2008), justifying the maintenance of the genera, despite being monotypical.

**Table 1.**
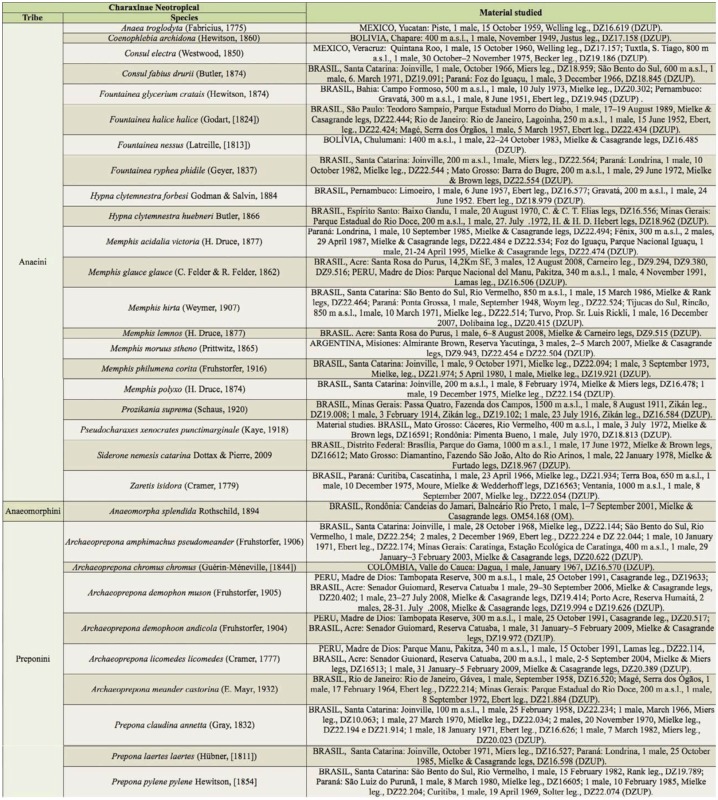
Species and materials studied.

**Figure 23.**
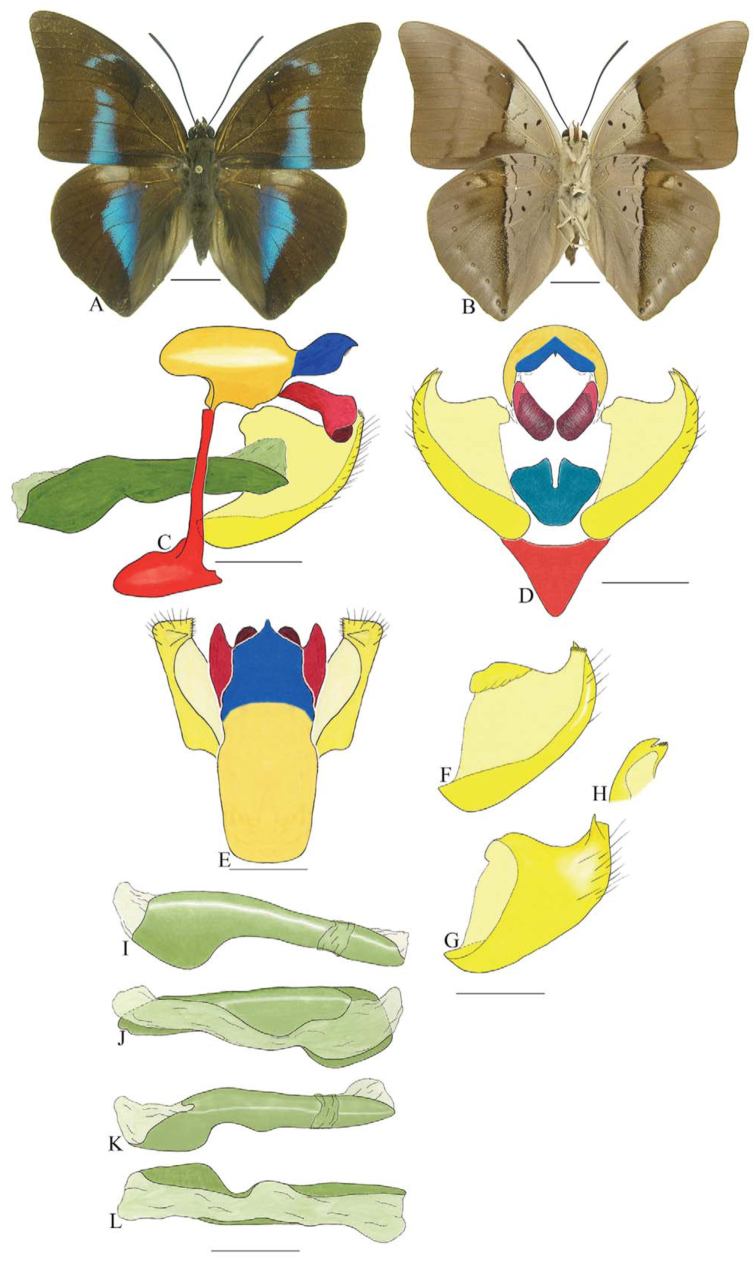
*Archaeoprepona amphimachus pseudomeander* (Fruhstorfer). A. Dorsal. B. Ventral. C–L Genitalia: C. Lateral view. D. Posterior view. E. Dorsal view. F–H Valva: F. Internal. G. External. H. Dorsal. I–L: Penis: I. Right lateral view. J. Left lateral view. K. Dorsal view. L. Ventral view. Scale bar: A and B = 1 cm. C–L = 1 mm. High quality figures are available online.

**Figure 24.**
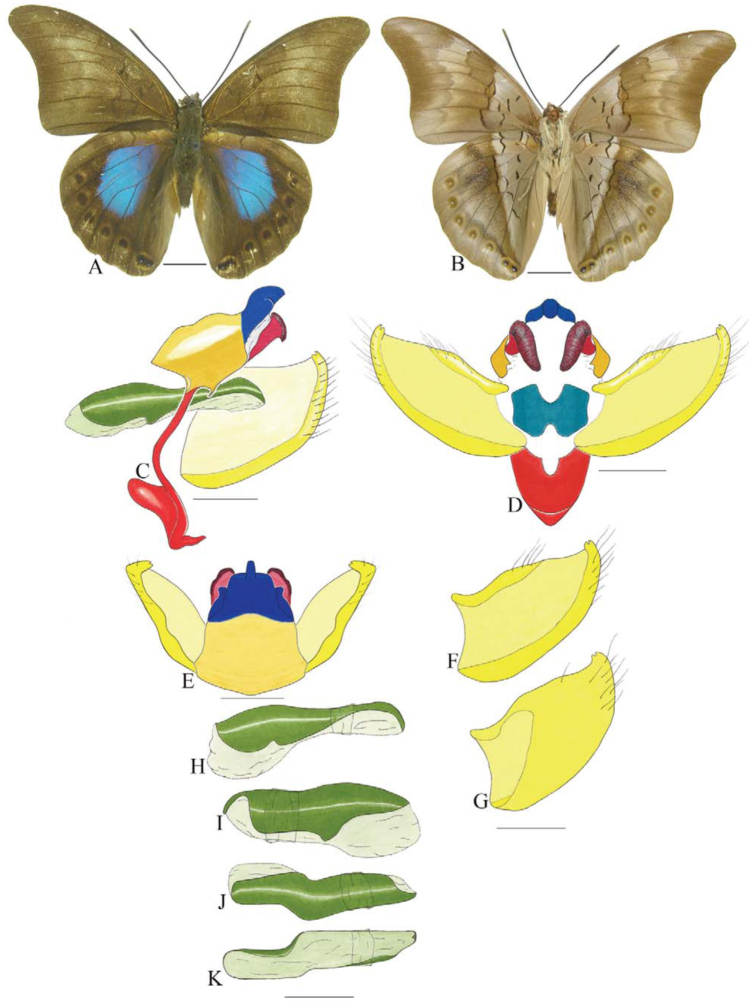
*Archaeoprepona chromus chromus* (Guérin-Méneville). A. Dorsal. B. Ventral. C–K Genitalia: C. Lateral view. D. Posterior view. E. Dorsal view. F and G Valva: F. Internal. G. External. H–K: Penis: H. Right lateral view. I. Left lateral view. J. Dorsal view. K. Ventral view. Scale bar: A and B = 1 cm. C–K = 1 mm. High quality figures are available online.

**Figure 25.**
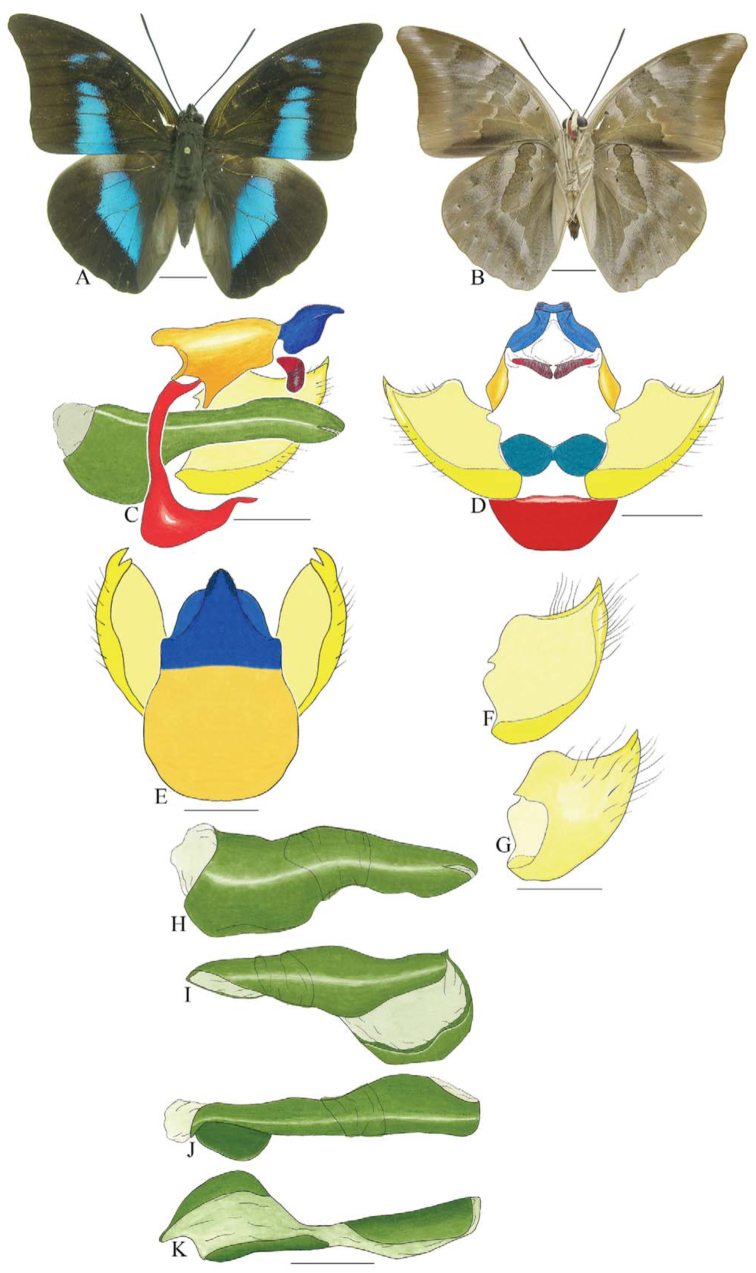
*Archaeoprepona demophon muson* (Fruhstorfer). A. Dorsal. B. Ventral. C–K Genitalia: C. Lateral view. D. Posterior view. E. Dorsal view. F and G Valva: F. Internal. G. External. H–K: Penis: H. Right lateral view. I. Left lateral view. J. Dorsal view. K. Ventral view. Scale bar: A and B= 1 cm. C–K= 1 mm. High quality figures are available online.

**Figure 26.**
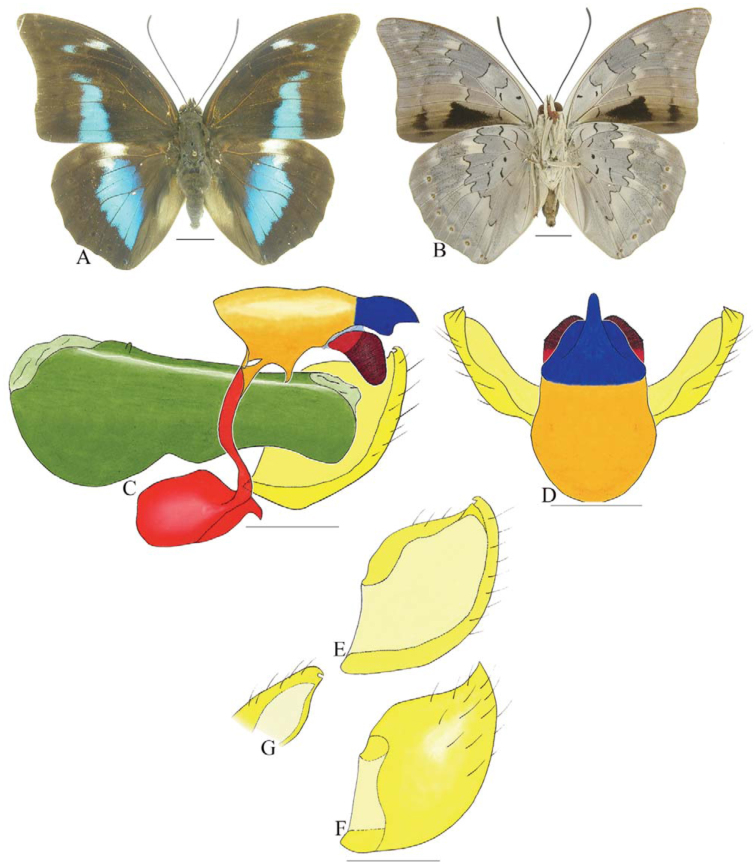
*Archaeoprepona demophoon andicola* (Fruhstorfer). A. Dorsal. B. Ventral. C–G Genitalia: C. Lateral view. D. Dorsal view. E–G Valva: E. Internal. F. External. G. Dorsal. Scale bar: A and B = 1 cm. C–G = 1 mm. High quality figures are available online.

**Figure 27.**
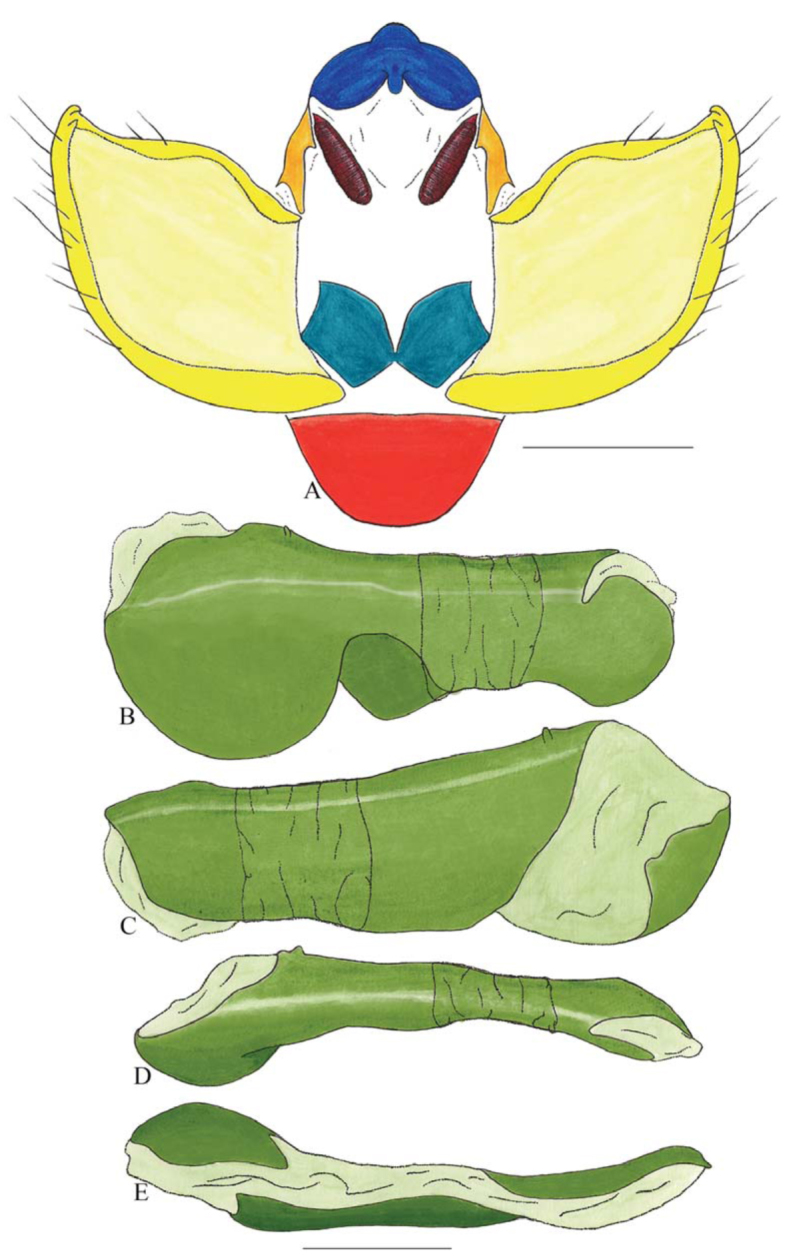
*Archaeoprepona demophoon andicola* (Fruhstorfer). A–E Genitalia: A Posterior. B–E Penis: B. Right lateral view. C. Left lateral view. D. Dorsal view. E. Ventral view. Scale bar: A–E= 1 mm. High quality figures are available online.

**Figure 28.**
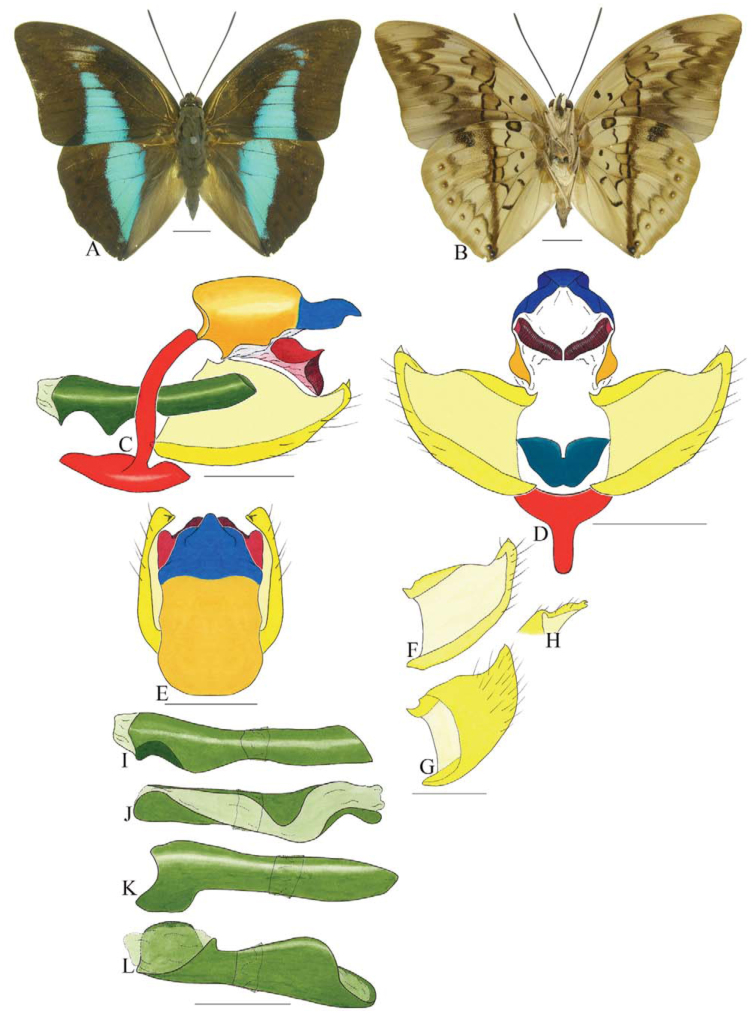
*Archaeoprepona licomedes licomedes* (Cramer). A. Dorsal. B. Ventral. C–L Genitalia: C. Lateral view. D. Posterior view. E. Dorsal view. F–H Valva: F. Internal. G. External. H. Dorsal. I–L: Penis: I. Right lateral view. J. Left lateral view. K. Dorsal view. L. Ventral view. Scale bar: A and B = 1 cm. C–L = 1 mm. High quality figures are available online.

**Figure 29.**
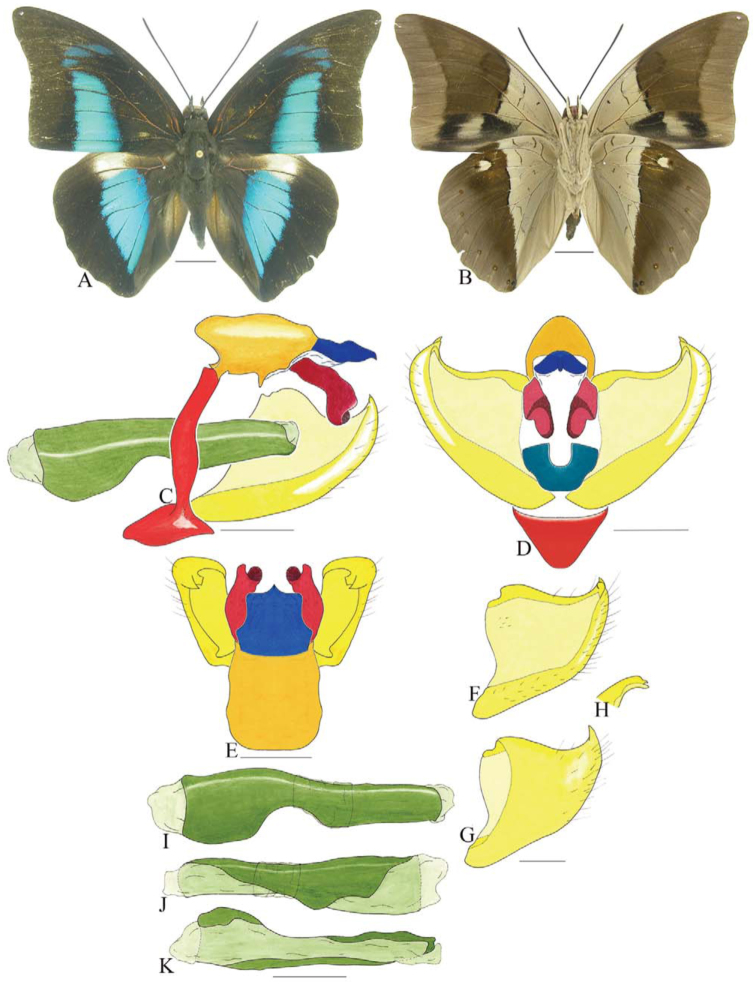
*Archaeoprepona meander meander* (Cramer). A. Dorsal. B. Ventral. C–K Genitalia: C. Lateral view. D. Posterior view. E. Dorsal view. F–H Valva: F. Internal. G. External. H. Dorsal. I–K: Penis: I. Right lateral view. J. Left lateral view. K. Ventral view. Scale bar: A and B = 1 cm. C–K = 1 mm. High quality figures are available online.

**Figure 30.**
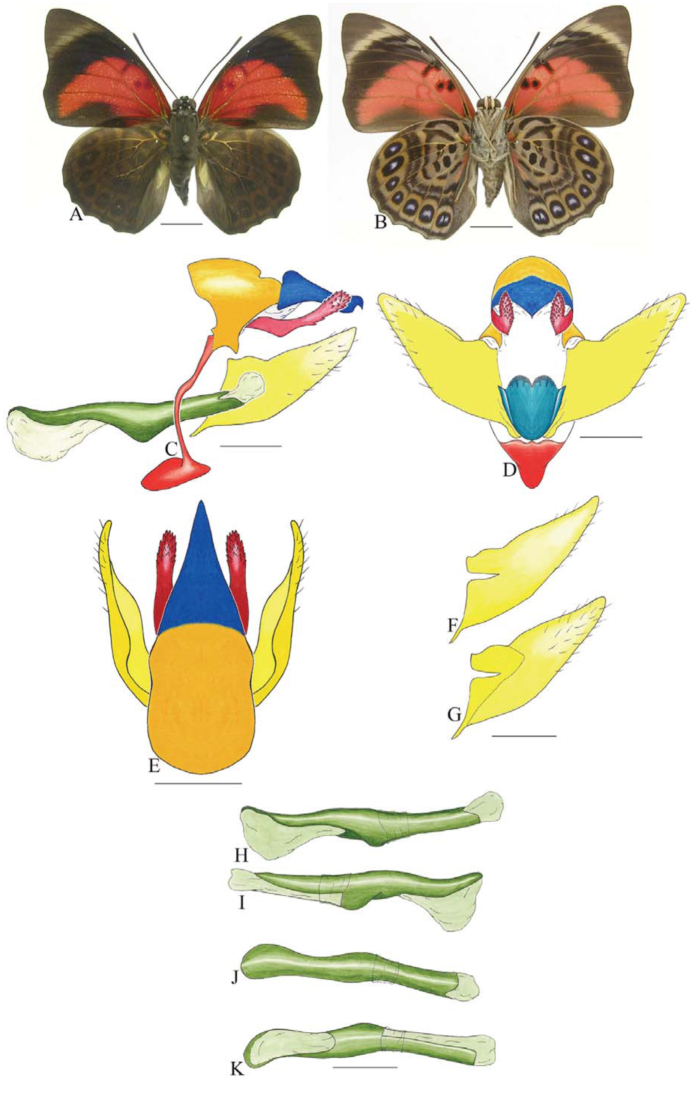
*Prepona claudina annetta* (Gray). A. Dorsal. B. Ventral. C–K Genitalia: C. Lateral view. D. Posterior view. E. Dorsal view. F and G Valva: F. Internal. G. External. H–K: Penis: H. Right lateral view. I. Left lateral view. J. Dorsal view. K. Ventral view. Scale bar: A and B = 1 cm. C–K = 1 mm. High quality figures are available online.

**Figure 31.**
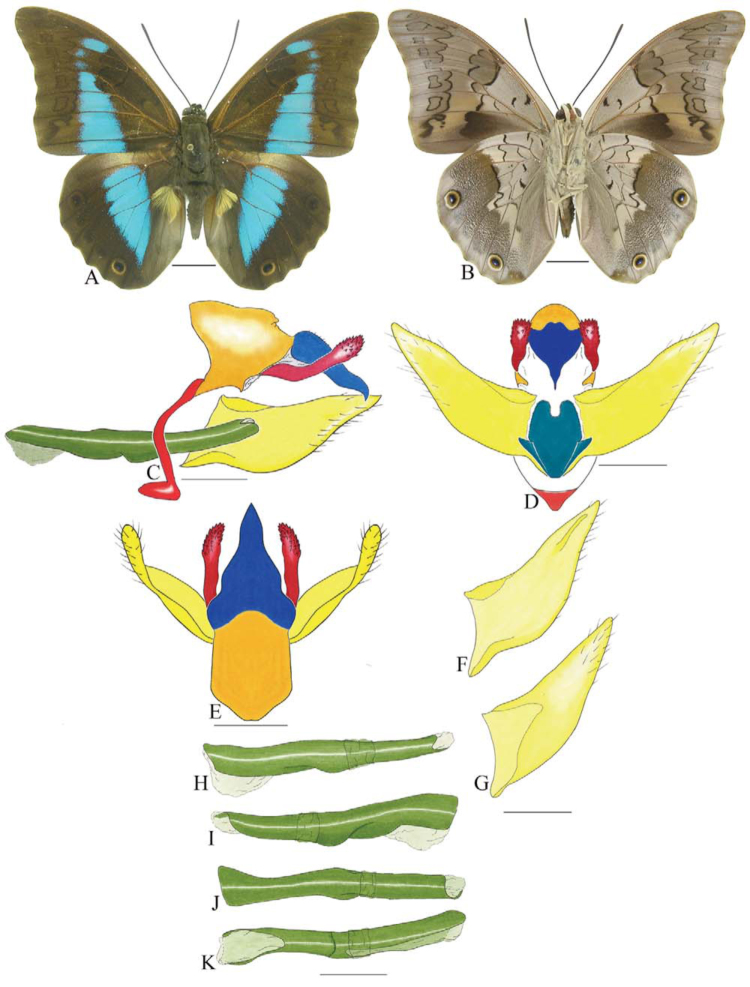
*Prepona laertes laertes* (Hubner). A. Dorsal. B. Ventral. C–K Genitalia: C. lateral view. D. Posterior view. E. Dorsal view. F and G Valva: F. Internal. G. External. H–K: Penis: H. Right lateral view. I. Left lateral view. J. Dorsal view. K. Ventral view. Scale bar: A and B = 1 cm. C–K = 1 mm. High quality figures are available online.

**Figure 32.**
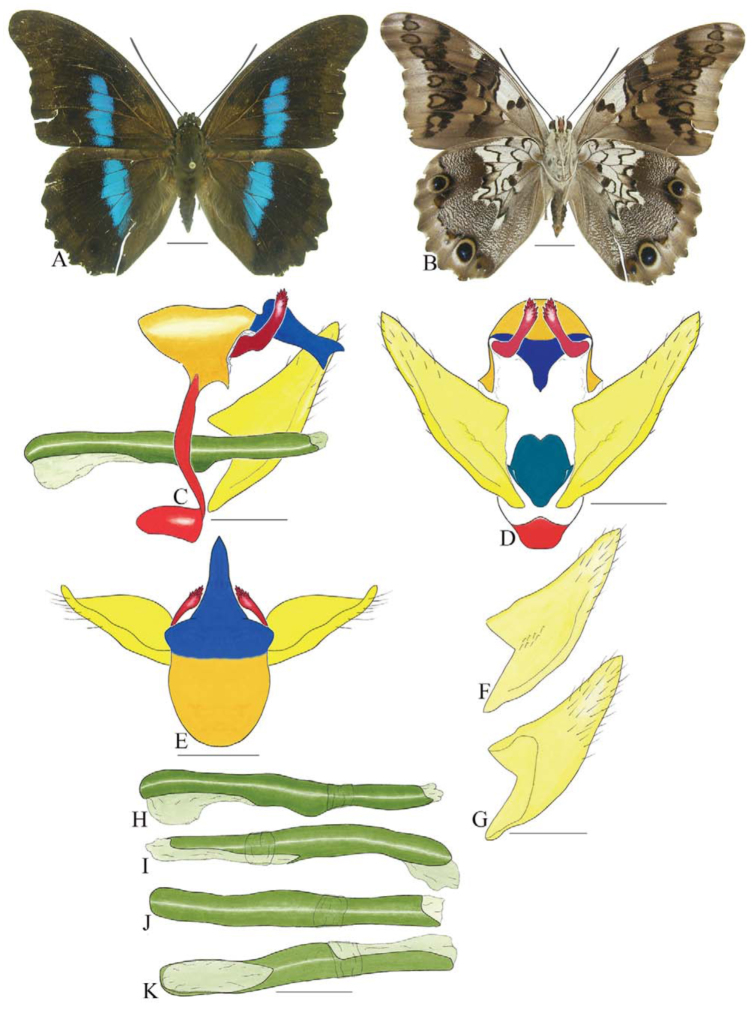
*Prepona pylene pylene* Hewitson. A. Dorsal. B. Ventral. C–K Genitalia: C. Lateral view. D. Posterior view. E. Dorsal view. F and G Valva: F. Internal. G. External. H–K: Penis: H. Right lateral view. I. Left lateral view. J. Dorsal view. K. Ventral view. Scale bar: A and B = 1 cm. C–K = 1 mm. High quality figures are available online.

**Figure 33.**
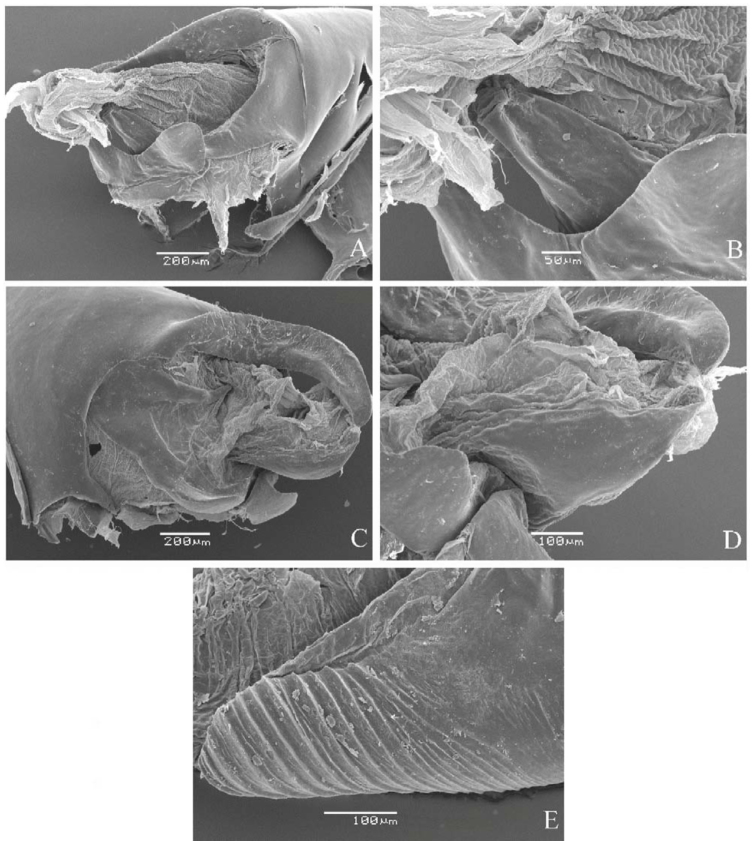
SEM photographs. A and B: Subscaphim of *Fountainea ryphea phidile.* C and D: Subscaphium of *Consul fabius drurii.* E. Stried gantho of *Archaeoprepona amphimachus pseudomeander.* High quality figures are available online.
